# DNA replication stress mediates APOBEC3 family mutagenesis in breast cancer

**DOI:** 10.1186/s13059-016-1042-9

**Published:** 2016-09-15

**Authors:** Nnennaya Kanu, Maria Antonietta Cerone, Gerald Goh, Lykourgos-Panagiotis Zalmas, Jirina Bartkova, Michelle Dietzen, Nicholas McGranahan, Rebecca Rogers, Emily K. Law, Irina Gromova, Maik Kschischo, Michael I. Walton, Olivia W. Rossanese, Jiri Bartek, Reuben S. Harris, Subramanian Venkatesan, Charles Swanton

**Affiliations:** 1 UCL Cancer Institute, CRUK Lung Cancer Centre of Excellence, Paul O’Gorman Building, Huntley St., London, UK; 2 Translational Cancer Therapeutics Laboratory, The Francis Crick Institute, London, UK; 3 Danish Cancer Society Research Center, Copenhagen, Denmark; 4 Department of Medical Biochemistry and Biophysics, Division of Translational Medicine and Chemical Biology, Karolinska Institute, Stockholm, Sweden; 5 CRUK Cancer Therapeutics Unit, The Institute of Cancer Research, London, UK; 6 Howard Hughes Medical Institute, Masonic Cancer Center, Institute for Molecular Virology, Center for Genome Engineering, Department of Biochemistry, Molecular Biology, and Biophysics, University of Minnesota, Minneapolis, Minnesota USA; 7 Department of Mathematics and Technology, University of Applied Sciences Koblenz, RheinAhrCampus Remagen, Joseph-Rovan-Allee 2, D-53424 Remagen, Germany

**Keywords:** APOBEC, Genomic instability, Replication stress, Somatic mutation

## Abstract

**Background:**

The APOBEC3 family of cytidine deaminases mutate the cancer genome in a range of cancer types. Although many studies have documented the downstream effects of APOBEC3 activity through next-generation sequencing, less is known about their upstream regulation. In this study, we sought to identify a molecular basis for APOBEC3 expression and activation.

**Results:**

*HER2* amplification and *PTEN* loss promote DNA replication stress and APOBEC3B activity *in vitro* and correlate with APOBEC3 mutagenesis *in vivo*. HER2-enriched breast carcinomas display evidence of elevated levels of replication stress-associated DNA damage *in vivo*. Chemical and cytotoxic induction of replication stress, through aphidicolin, gemcitabine, camptothecin or hydroxyurea exposure, activates transcription of *APOBEC3B* via an ATR/Chk1-dependent pathway *in vitro*. APOBEC3B activation can be attenuated through repression of oncogenic signalling, small molecule inhibition of receptor tyrosine kinase signalling and alleviation of replication stress through nucleoside supplementation.

**Conclusion:**

These data link oncogene, loss of tumour suppressor gene and drug-induced replication stress with APOBEC3B activity, providing new insights into how cytidine deaminase-induced mutagenesis might be activated in tumourigenesis and limited therapeutically.

**Electronic supplementary material:**

The online version of this article (doi:10.1186/s13059-016-1042-9) contains supplementary material, which is available to authorized users.

## Significance

APOBEC3 mutates the cancer genome in a broad range of cancer types. In this study we link DNA replication stress mediated by oncogene activation or cytotoxic exposure to APOBEC3B activity. These insights provide support for therapeutic approaches that might limit the activity of this mutagenic process.

## Background

Genomic instability is a well-recognized hallmark of cancer and is known to cause both aberrant chromosome architecture as well as mutational changes at the single nucleotide level [1]. We have previously identified a role for DNA replication stress in human tumourigenesis [2, 3] and in the generation of chromosomal instability, which contributes to intratumour heterogeneity [4, 5]. More recently, analyses performed in over 30 cancer types have identified that many tumours exhibit cytosine mutation biases, particularly C to T transitions and C to G transversions predominantly in T*C*A or T*C*T trinucleotide contexts [6–9]. The mutagen has been identified as a member of the apolipoprotein B mRNA editing enzyme, catalytic polypeptide-like 3 (APOBEC3) family of cytidine deaminases [9–11]. We recently described the enrichment of APOBEC3 mutagenesis later in tumour evolution, occurring as subclonal mutations in estrogen receptor (ER)-negative breast cancer, lung adenocarcinoma, head and neck squamous carcinoma and bladder carcinoma, suggesting APOBEC3 may contribute to branched evolution in some tumour types [12–14].

Although, the involvement of APOBEC3 in cancer has been refined over the past few years, the functional regulation of this family of enzymes is yet to be fully understood. A closer examination of kataegis in cancer samples revealed that APOBEC3-induced mutations often colocalised with breakpoint rearrangements, and within breast cancer, the HER2-enriched (HER2+) subtype has been shown to display evidence of APOBEC3-mediated mutagenesis [9]. Furthermore, HER2+ breast cancer is associated with high levels of somatic copy number aberrations (SCNAs) [9]. Whether there is a mechanistic connection between the underlying causes of chromosomal copy number aberrations and the generation of APOBEC3 mutagenesis in HER2+ breast cancer has not been explored.

The extent of hypermutation is likely to be dependent on both the level of APOBEC3 protein and the availability of single-stranded DNA (ssDNA) substrate [9]. In the presence of cellular cytidine deaminase, however, the rate-limiting step is thought to be substrate availability [15]. It is thought that segmental SCNA breakpoints could potentially expose more ssDNA, which is the ideal substrate for APOBEC3 [15, 16]. The availability of ssDNA substrate can be modulated by regulating replication fork stability and collapse [17]. Additional processes that induce ssDNA exposure include oncogene-induced replication stress, double strand break (DSB) repair [18], R-loops formed during transcription [19] and telomere crisis [20]. ssDNA can also be exposed by DNA end resection during DSB repair [15]. In addition, Gordenin and colleagues [16] previously identified that the extent of strand-coordinated mutation clusters in yeast was increased following exposure of cells to the chemical mutagen methyl-methanesulfonate.

In this study, we investigated the genomic correlates of APOBEC3 mutagenesis in breast cancer. We examined whether DNA damage signalling, triggered by ssDNA exposure by cytotoxic agents or oncogenic signalling, may contribute to APOBEC3 activation and the mutational signature profile seen in breast cancer.

## Results

### HER2 amplification, *PTEN* and *NF1* somatic mutations are associated with the APOBEC3 signature

**Fig. 1:**
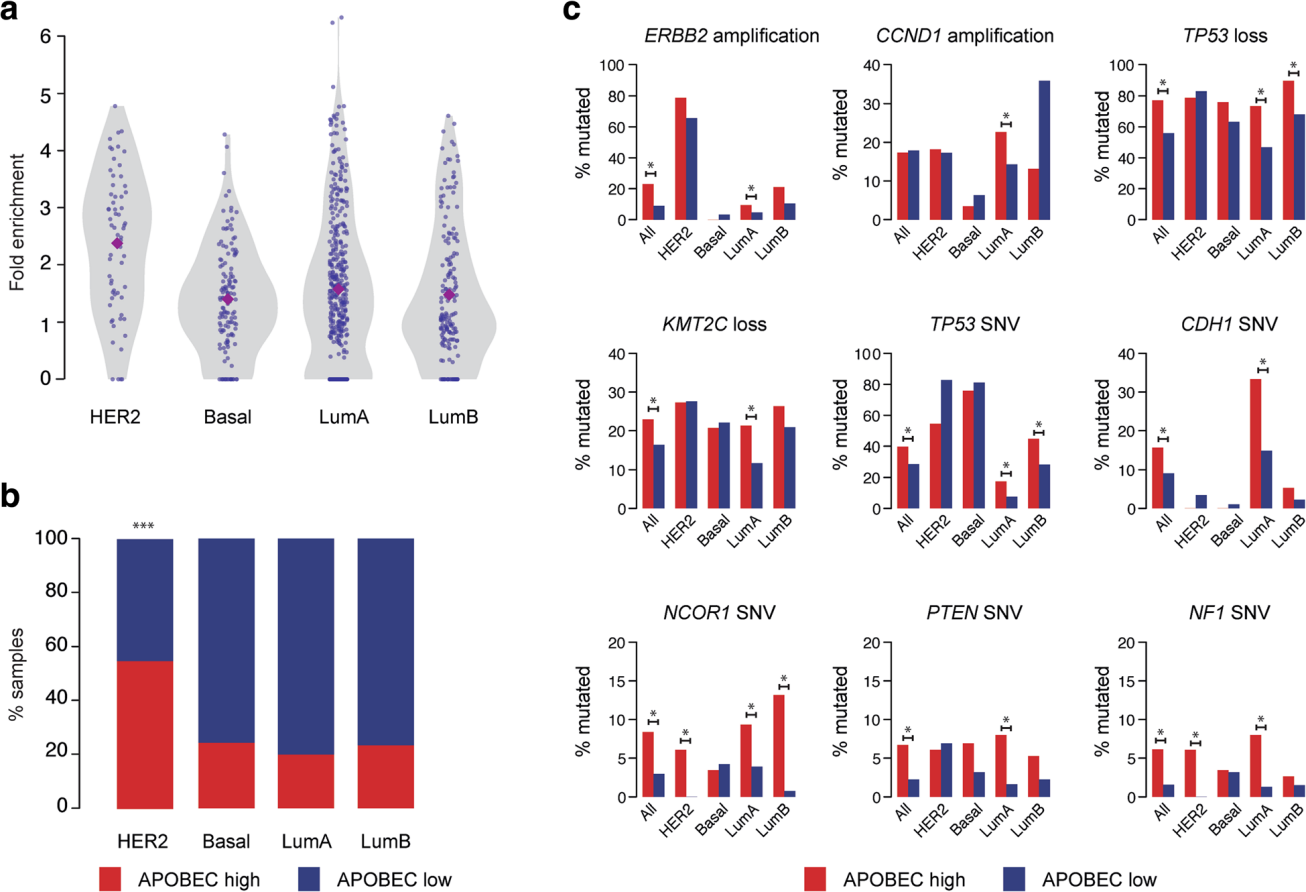
APOBEC3 mutational signatures and associated genes in breast cancer subtypes. **a** Violin plots showing APOBEC3 mutagenesis fold enrichment. The *purple diamond* represents the median in each subtype. **b** Boxplots showing percentage of ‘APOBEC high’ (*red*) and ‘APOBEC low’ (*blue*) samples in each subtype. *Asterisks* represent a significant *p* value <0.05 from pairwise post hoc tests. **c** Single-nucleotide variants (*SNVs*) and SCNAs associated with ‘APOBEC high’ tumour samples. *Bars* denote proportion of ‘APOBEC high’ (*red*) and ‘APOBEC low’ (*blue*) samples harbouring mutation. Amplification and loss refer to ≥2× ploidy and ≤1 copy number relative to ploidy, respectively. An *asterisk* denotes significant association in subtype (q < 0.1 by permutation test, corrected for analysis of multiple genes by the Benjamini–Hochberg method). Note differing scales used on the *y-axis. Lum* luminal

It has recently been shown that HER2-enriched (HER2+) breast cancers are associated with a high burden of mutations attributable to APOBEC3B [9]. We utilized breast cancer samples from The Cancer Genome Atlas (TCGA; *n* = 755) [21], which were subclassified using the PAM50 algorithm into HER2+, basal, luminal A and luminal B subtypes [22], and assessed the fold enrichment of APOBEC3 signature mutations in each sample. Consistent with a previous report [9], the APOBEC3 mutagenesis pattern was significantly associated with the HER2+ subtype (*p* value = 1.086 × 10^−5^, chi-square test; Fig. 1a, b). We also observed that HER2 amplification was significantly associated with ‘APOBEC high’ samples in the luminal A subtype (false discovery rate (FDR) q-value = 0.075, permutation test; see “Methods”), implicating HER2 as a driver of APOBEC3 mutagenesis in this subtype (Fig. 1c). Additionally, mutations in *TP53*, *CDH1*, *NCOR1*, *PTEN* and *NF1*, *CCND1* amplification, as well as loss of *TP53* and *KMT2C* were associated (FDR q-value <0.1, permutation test) with ‘APOBEC high’ samples in different breast cancer subtypes (Fig. 1c), which could explain the heterogeneity in APOBEC3 enrichment among samples within subtypes. Mutations in *PIK3CA* were also associated with the APOBEC3 signature, although it has been suggested that APOBEC3 activity itself is the main driver of these helical domain mutations [23]. We further observed that ‘APOBEC high’ tumours had a higher number of segmental SCNA breakpoints per sample compared with ‘APOBEC low’ tumours (*p* value = 0.000343, Mann–Whitney U test; Additional file 1: Figure S1a).

**Fig. 2:**
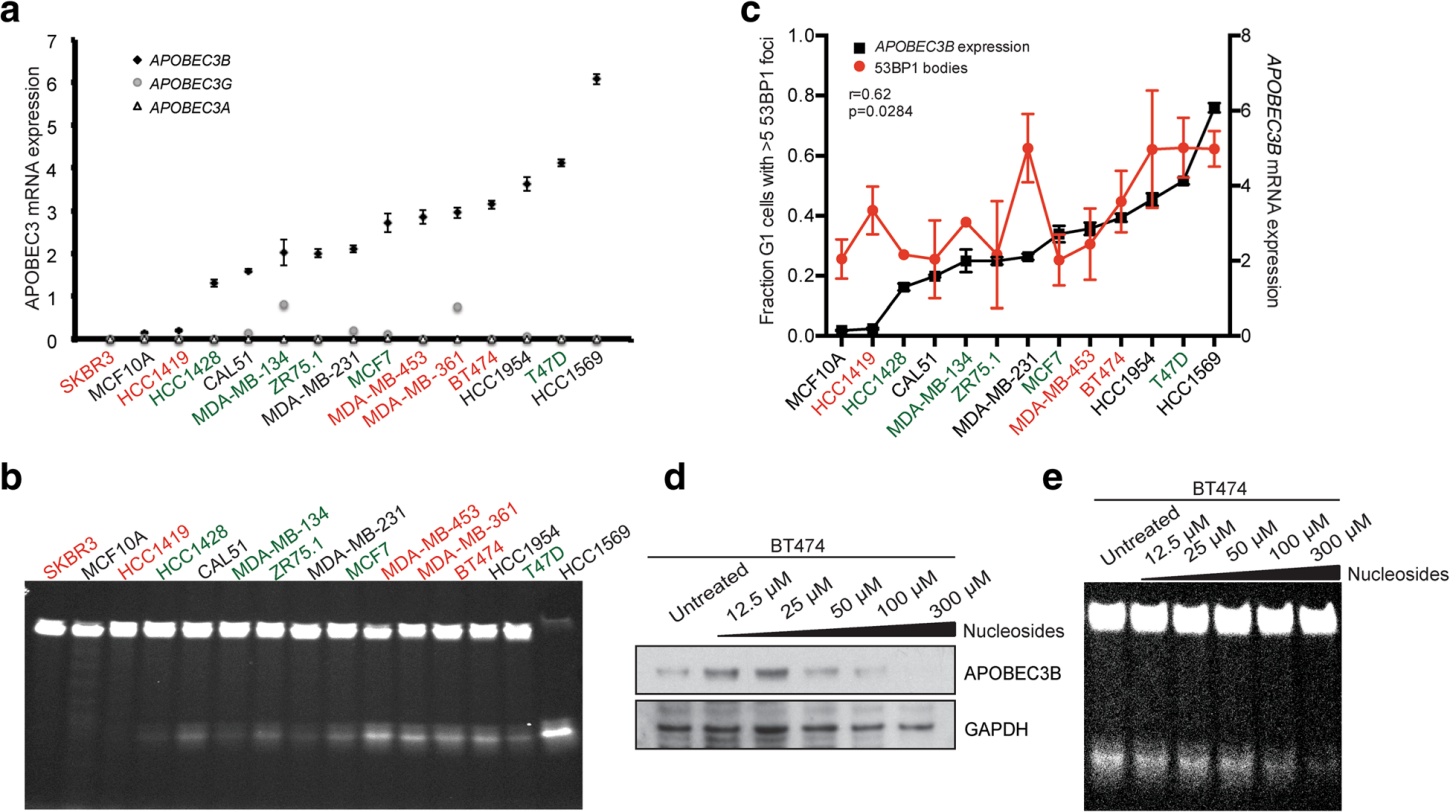
APOBEC3 activity and replication stress in breast cancer cell lines. **a** *APOBEC3B* (*black*), *APOBEC3G* (*grey*) and *APOBEC3A* (*white*) mRNA expression in 15 breast cancer cell lines as determined by quantitative PCR. HER2+ cell lines (*red*), basal cell lines (*black*), luminal cell lines (*green*). SKBR3 cells have a null mutation for *APOBEC3B*. Error bars represent standard deviation. **b** APOBEC3 activity in the 15 breast cancer cell lines used in **a**. Cells were lysed and subjected to oligonucleotide-based cytidine deamination assay followed by electrophoresis on 15 % TBE-urea gels. **c** Cells were grown for two population doublings on glass coverslips followed by fixation and staining with 53BP1 and cyclin A antibodies. The fractions of cyclin A-negative cells displaying more than five 53BP1 nuclear foci were scored. *APOBEC3B* mRNA expression was determined by quantitative PCR from parallel cell lysates. A Spearman’s rank correlation test was performed to correlate the fraction of 53BP1 nuclear bodies in cell lines with the level of *APOBEC3B* (r = 0.62, *p* = 0.0284). Error bars represent standard deviation. **d** BT474 cells were treated with 12.5–300 μM nucleosides for 72 h prior to lysis. Western blots were probed with the indicated antibodies. **e** BT474 cells were treated as in **d** followed by lysis and an APOBEC3 cytidine deamination assay

We examined *APOBEC3A*, *APOBEC3B* and *APOBEC3G* mRNA expression levels in a panel of 15 breast cancer cell lines (five luminal, five basal and five HER2+) by quantitative PCR (Fig. 2a). Most luminal cell lines (green) exhibited low levels of *APOPEC3B* mRNA expression, whereas most of the HER2+ (red) exhibited higher *APOBEC3B* mRNA levels (Fig. 2a). Basal cell lines (black) exhibited variable *APOBEC3B* mRNA levels (Fig. 2a). *APOBEC3B* expression was undetectable in SKBR3 cells, which are known to have a homozygous deletion of *APOBEC3B*. The basal mRNA expression of *APOBEC3A* and *APOBEC3G* was almost undetectable in all cell lines tested (Fig. 2a). The observed mRNA expression levels were comparable to those identified in the Cancer Cell Line Encyclopedia (CCLE) dataset (Additional file 1: Figure S1b). We also examined the deamination activity present in these cell lysates determined using an oligonucleotide-based cytidine deamination assay [10] using two probes whose activity is dependent on APOBEC3B (Fig. 2b; Additional file 1: Figure S1c–f). There was a significant correlation between *APOBEC3B* expression and activity in these cell lines (r = 0.8, *p* = 0.0016, Spearman rank correlation test; Additional file 1: Figure S1g).

Since HER2 signalling is known to drive a proliferative phenotype, which might provoke elevated DNA replicative stress (reviewed in [24]), we next investigated markers of DNA replicative stress in the 15 breast cancer cell lines. Immunofluorescence analysis using 53BP1 nuclear bodies in G1 as a marker of DNA replication stress [25] revealed that cell lines with higher levels of *APOBEC3B* expression had significantly higher levels of replication stress (r = 0.62, *p* = 0.0284, Spearman rank correlation test; Fig. 2c; Additional file 1: Figure S1h). SKBR3 (*APOBEC3B* null) and MDA-MB-361 (with a missense mutation in *53BP1*) cell lines were both excluded from the analysis. To further confirm the relevance of DNA replication stress in APOBEC3 activation, we investigated whether alleviating replication stress would reduce APOBEC3 activity. Previously, it has been shown that supplementation of exogenous nucleosides increases the nucleotide pool in the cell and can rescue replication stress [26, 27]. Therefore, we treated BT474 cells, a HER2-amplified cell line with elevated DNA replication stress (Fig. 2c), with exogenous nucleosides prior to performing the oligonucleotide-based deamination assay. Treatment with 12.5–300 μM nucleosides for 72 h led to a significant reduction in the basal levels of APOBEC3B protein and activity in a dose-dependent manner (Fig. 2d, e). Supplementation of MDA-MB-134 cells (a luminal cell line with low but detectable APOBEC3 activity) with exogenous nucleosides also led to a reduction in basal APOBEC3 activity (Additional file 2: Figure S2d). No correlation was observed between reductions in cell viability in response to 300-μM nucleoside treatment (Additional file 1: Figure S1i; Additional file 3: Figure S3b) and the induction of APOBEC3 activity. Nucleoside supplementation reduced the S phase population in MDA-MB-134 cells but had minimal effect on the cell cycle distribution of BT474 cells (Additional file 1: Figure S1j; Additional file 4: Figure S4d). Taken together these results implicate the involvement of DNA replication stress in APOBEC3-mediated mutagenesis.

### Replication stress induced by cytotoxic drugs leads to APOBEC3 induction

**Fig. 3:**
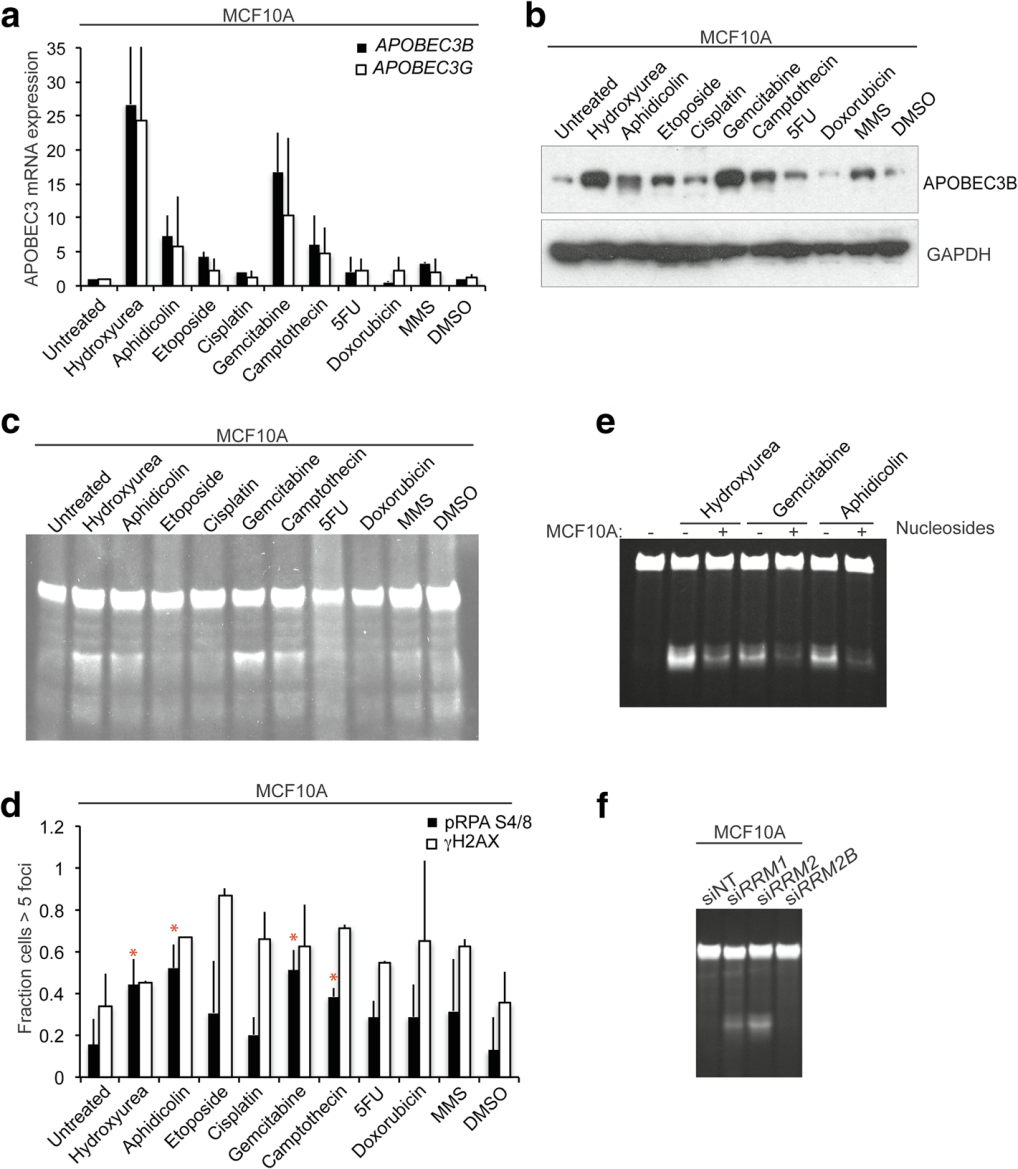
Induction of replication stress and APOBEC3 activity in breast cancer cell lines. **a** MCF10A cells were treated with the indicated drugs for 48 h followed by mRNA extraction, cDNA synthesis and quantitative PCR for *APOBEC3B* and *APOBEC3G* expression levels. **b** MCF10A cells were treated as in **a** followed by western blotting with the indicated antibodies. **c** MCF10A cells were treated as in **a** prior to lysis and a cytidine deamination assay for APOBEC3 activity using probe 2. **d** MCF10A cells were treated as in **a** followed by fixation and immunofluorescence for Ser139 γH2AX and S4/8 replication protein A phosphorylation (*pRPA*). *Red asterisks* indicate treatments inducing *APOBEC3B* mRNA, protein expression, activity levels and S4/8 RPA phosphorylation. **e** MCF10A cells were pre-treated with 300 μM exogenous nucleosides followed by incubation with the indicated drugs for an additional 24 h. Following lysis, APOBEC3 activity was measured by a cytidine deamination assay. **f** Ribonucleotide reductase subunits *RRM1*, *RRM2* and *RRM2B* were depleted from MCF10A cells by RNA interference and, after 72 h, cells were lysed and subjected to an APOBEC3 cytidine deamination assay. *5FU* 5-fluorouracil, *MMS* methyl methanesulfonate, *siNT* non-targeting control siRNA

In order to decipher the mechanism through which replication stress is implicated in the induction of APOBEC3 activity, we tested a panel of cytotoxic drugs known to induce either DSB or ssDNA damage. MCF10A cells were treated with nine drugs with broad DNA damaging or anti-metabolite activity (hydroxyurea, aphidicolin, cisplatin, gemcitabine, etoposide, camptothecin, methylmethanesulfonate, doxorubicin and 5-fluorouracil) for 48 h, after which APOBEC3 mRNA expression, protein and activity levels were assessed. Treatment of MCF10A cells with hydroxyurea, aphidicolin, gemcitabine and camptothecin elicited an increase in both *APOBEC3B* and *APOBEC3G* mRNA expression (Fig. 3a), APOBEC3B protein expression (Fig. 3b) and APOBEC3 activity (Fig. 3c; Additional file 2: Figure S2a; Additional file 5: Figure S5). Treatment of MCF7, HCC1419 and MDA-MB-134 cells with hydroxyurea, aphidicolin and gemcitabine also led to an increase in APOBEC3 activity (Additional file 2: Figure S2b–d). SKBR3 cells were included as a negative control (Additional file 2: Figure S2e). By performing the cytidine deamination assays following depletion of *APOBEC3B* by RNA interference (RNAi), we confirmed that all detectable hydroxyurea-induced deamination activity in the breast cancer cell lines was attributable to *APOBEC3B* (Additional file 2: Figure S2f, g). No correlation was observed between drug-induced cytotoxity (Additional file 3: Figure S3a–d) and APOBEC3 activity. We observed that the four cytotoxic drugs that elicited the highest levels of APOBEC3B induction were associated with S phase enrichment in HCC1419 and MDA-MB-134 cells. Cell cycle arrest in MCF10A cells was also associated with an accumulation of cells at G2/M (Additional file 4: Figure S4).

In order to investigate the type of DNA damage induced by drug exposure, we assessed the extent of DSBs and ssDNA damage caused by these drugs by immunofluorescence staining of Ser139 γH2AX and pS4/8 replication protein A (RPA), respectively (Fig. 3d; Additional file 6: Figure S6a). There was a significant correlation between the drugs that caused the highest APOBEC3B induction and the induction of RPA phosphorylation in MCF10A cells (Additional file 6: Figure S6b–d). The four drugs that induced APOBEC3B activity all induced the highest levels of RPA phosphorylation in MCF10A cells (Fig. 3d), whereas in MCF7 cells this was only the case for three out of the four drugs (Additional file 6: Figure S6a). There was no correlation between drugs inducing DSBs and APOBEC3 induction. Furthermore, we observed that exposure of MCF10A cells to exogenous nucleosides also attenuated the hydroxyurea, aphidicolin and gemcitabine-induced increase in APOBEC3 activity (Fig. 3e). Nucleoside supplementation reduced the hydroxyurea-induced S phase enrichment in MCF10A cells (Additional file 3: Figure S3e). These results suggest that DNA replication stress is able to increase APOBEC3 transcription levels and trigger its activity.

Hydroxyurea is an inhibitor of ribonucleotide reductase, an enzyme that catalyses the reduction of ribonucleotides to deoxyribonucleotides, which are required for DNA replication. Depletion of ribonucleotide reductase stalls the DNA polymerase at the replication forks, resulting in DNA replication stress [28]. In order to further confirm the role for DNA replication stress in APOBEC3 regulation, we depleted ribonucleotide reductase subunits *RRM1*, *RRM2* and *RRM2B* by small interfering RNA (siRNA) in MCF10A cells since they exhibit low levels of basal APOBEC3B activity and replication stress. Knockdown of either *RRM1* or *RRM2* subunits by siRNA led to an increase in APOBEC3B deamination activity (Fig. 3f; Additional file 6: Figure S6e). Knockdown of *RRM2B* encoding the small subunit of p53 inducible ribonucleotide reductase had no effect. These results suggest a relationship between the induction of ssDNA and APOBEC3B induction.

### HER2 expression and *PTEN* knockdown contribute to APOBEC3 activity

**Fig. 4:**
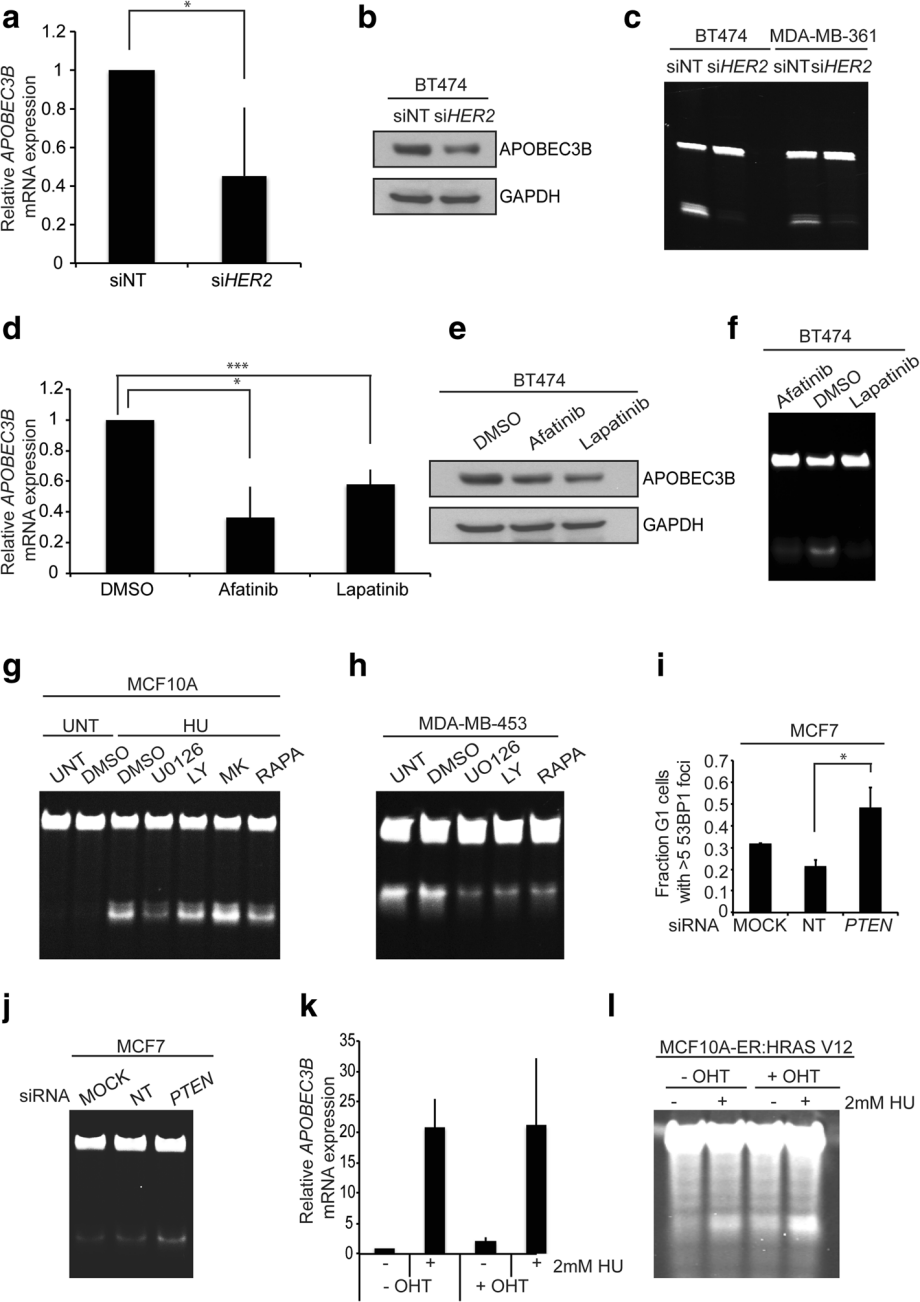
HER2 expression and *PTEN* contribute to APOBEC3 activity. **a** *APOBEC3B* mRNA expression following silencing of HER2 expression in BT474 cells by RNAi. HER2 levels were depleted by RNAi and, after 72 h, cells were harvested and mRNA extracted. Following cDNA synthesis, *APOBEC3B* mRNA levels were determined by quantitative PCR; *p < 0.01 (*t*-test). *siNT* non-targeting control siRNA. **b** BT474 cells were treated as in **a** and, following lysis, western blots were probed with the indicated antibodies. **c** BT474 and MDA-MB-361 cells were treated as in **a** and, following lysis, samples were subjected to cytidine deamination assay to determine levels of APOBEC3 activity. **d** BT474 cells were treated with 10 nM afatinib or 30 nM lapatinib for 24 h followed by mRNA isolation and quantitative PCR to determine *APOBEC3B* mRNA expression levels; *p < 0.01, ***p < 0.005 (*t*-test). **e** BT474 cells were treated as in **d** and, following lysis, western blots were probed with the indicated antibodies. **f** BT474 cells were treated as in **d** and, following lysis, samples were subjected to cytidine-based deamination assay to determine levels of APOBEC3 activity. **g** MCF10A cells were treated with or without 2 mM hydroxyurea (*HU*) and exposed to the indicated drugs for 48 h followed by APOBEC3 cytidine deamination assay. **h** MDA-MB-453 cells were treated with the indicated drugs for 48 h followed by APOBEC3 cytidine deamination assay. **i** PTEN levels were depleted from MCF7 cells growing on glass coverslips by RNAi. Cells were fixed and stained with 53BP1 and cyclin A antibodies. The fraction of cyclin A-negative cells displaying more than five 53BP1 nuclear foci were scored; *p < 0.05 (*t*-test). **j** PTEN levels were depleted from MCF7 cells by RNAi. After 72 h cells were harvested and samples were subjected to cytidine deamination assay to determine APOBEC3 activity. **k** APOBEC3 activity in response to RAS induction and hydroxyurea (*HU*) treatment. MCF10A-ER:HRAS V12 cells were induced with tamoxifen (4-hydroxytamoxifen; *4-* *OHT*) in either the presence or absence of hydroxyurea for 48 h, followed by mRNA isolation, cDNA synthesis and quantitative PCR to determine *APOBEC3B* expression levels. **l** MCF10A-ER:HRAS V12 cells were treated as in **k**. Cells were subsequently lysed and subjected to APOBEC3 cytidine deamination assay. *LY* LY294002, *MK* MK2206, *NT* non-targeting, *RAPA* rapamycin, *UNT* untreated

Having observed increased replication stress and APOBEC3 activity in many of the HER2+ cell lines, we next investigated the consequence of HER2 depletion on APOBEC3 activity using the oligonucleotide-based deamination assay. Silencing HER2 by RNAi in HER2+ BT474 and MDA-MB-361 cells led to a reduction in *APOBEC3B* mRNA expression, protein levels and deamination activity (Fig. 4a–c; Additional file 7: Figure S7a). To further examine whether this reduction was dependent on the presence of HER2 protein or on HER2 downstream signalling, *APOBEC3B* mRNA expression, protein and activity levels were assessed following exposure of HER2+ cells to HER2 tyrosine kinase inhibitors. A 24-h treatment of BT474 cells with 10 nM afatinib and 30 nM lapatinib resulted in reduced *APOBEC3B* mRNA expression (by 64 % and 42 %, respectively), protein and deamination activity (Fig. 4d–f; Additional file 7: Figure S7b). There was no correlation between treatment-induced cytotoxicity and APOBEC3 induction (Additional file 8: Figure S8a). Treatment with lapatinib reduced the S phase population, whereas afatinib did not significantly alter the cell cycle distribution of BT474 cells (Additional file 8: Figure S8c). In addition, lapatinib treatment was able to reduce hydroxyurea-induced *APOBEC3B* transcription and activity in HER2+ HCC1419 (Additional file 7: Figure S7c–f). These results suggest that signal transduction cascades downstream of HER2 could be implicated in APOBEC3 induction.

We next investigated the requirement for phosphatidylinositol 3-kinase (PI3K), mitogen-activated protein kinase (MAPK), AKT and mammalian target of rapamycin (mTOR) signalling pathways on replication stress-induced APOBEC3 activation. First MCF10A cells, with a low basal level of replication stress, were treated with hydroxyurea in the presence of inhibitors of these signalling pathways for 24 h. Inhibition of MEK signalling (with U0126) and to a lesser extent mTOR signalling (with rapamycin) attenuated hydroxyurea-induced *APOBEC3G* transcription and APOBEC3 activity (Fig. 4g; Additional file 8: Figure S8d), implicating both arms of the signalling cascade in the exacerbation of DNA replication stress-induced APOBEC3 mutagenesis. Consistent with this finding, treatment of MDA-MB-453, HCC1569 and BT474 cells with U0126, rapamycin and LY294002 also led to a reduction in basal APOBEC3 activity (Fig. 4h; Additional file 8: Figure S8e, f). There was no correlation between the extent of cytotoxicity of the drugs and their ability to induce APOBEC3 activity (Additional file 8: Figure S8b; Additional file 9: Figure S9a). In BT474 cells treatment with UO126 and rapamycin appeared to reduce the G2/M phase (Additional file 8: Figure S8c). In MCF7 cells hydroxyurea appeared to induced cell cycle arrest in the S and G2/M phases, which could be rescued with rapamycin treatment (Additional file 9: Figure S9b). Since we identified that *PTEN* mutations were also associated with enrichment of the APOBEC3 mutational signature, we also investigated the effect of *PTEN* loss on replication stress-induced APOBEC3 activity. MCF7 cells were depleted of *PTEN* by siRNA and replication stress was assessed by scoring the presence of G1 nuclear bodies. Silencing of *PTEN* led to a significant increase in G1 bodies from 21 to 48 % (*p* value = 0.027, *t*-test; Fig. 4i) and an increase in APOBEC3B protein and cytidine deamination activity (Fig. 4j; Additional file 9: Figure S9c, d). *PTEN* knockdown did not cause a significant change in cell viability or a change in cell cycle distribution that could account for the increase in APOBEC3 activity observed (Additional file 9: Figure S9e, f).

### Oncogene-induced replication stress and APOBEC3 activation

Overexpression of several oncoproteins, including RAS, MYC, CCND1 and CCNE, has been demonstrated to cause increased origin firing and increased proliferation by accelerating G1/S transition leading to replication stress [3, 29–32]. To determine if oncogene-induced replication stress would also lead to APOBEC3 activation, we treated an MCF10A cell line stably expressing a 4-hydroxytamoxifen-inducible oncogenic RAS chimeric protein, ER:HRAS V12 [33], with hydroxyurea and assessed APOBEC3 activation. Treatment of MCF10A-ER:HRAS V12 cells with 2 mM hydroxyurea increased *APOBEC3B* mRNA expression approximately 20-fold, which was not further increased by the activation of RAS V12 (Fig. 4k). RAS V12 induction in the absence of hydroxyurea led to a modest increase in APOBEC3 deamination activity relative to non-induced cells (Fig. 4l). RAS V12 induction in the presence of hydroxyurea also led to a modest increase in APOBEC3 deamination activity compared with hydroxyurea treatment alone. These results illustrate that RAS hyperactivation alone is insufficient to significantly activate APOBEC3 in this system.

### ATR pathway implicated in APOBEC3 regulation

**Fig. 5:**
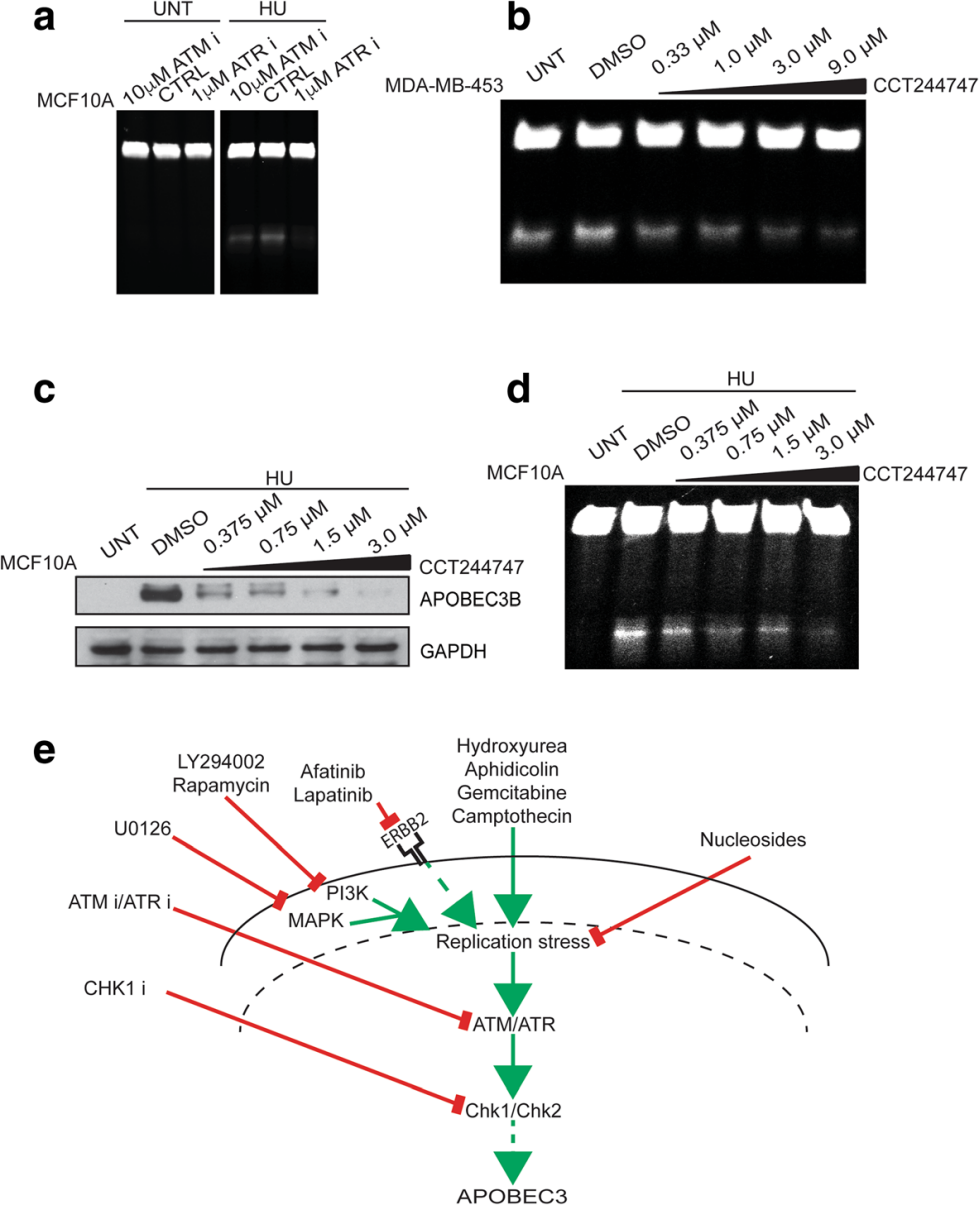
DNA damage signalling and APOBEC3 activity in breast cancer cell lines. **a** MCF10A cells were treated with ATM or ATR inhibitors for 24 h after which cells were treated with hydroxyurea (*HU*) for a further 48 h prior to lysis and cytidine deamination assay for APOBEC3 activity. **b** MDA-MB-453 cells were treated for 48 h with the indicated doses of Chk1 inhibitor CCT244747 followed by APOBEC3 cytidine deamination assay. **c** MCF10A cells were treated for 48 h with the indicated doses of Chk1 inhibitor CCT244747 and with 2 mM hydroxyurea for the last 24 h. Following lysis, western blots were probed with the indicated antibodies. **d** MCF10A cells were treated as in **c** before lysis and cytidine deamination assay to determine APOBEC3 activity. **e** Model illustrating mechanisms of APOBEC3 regulation by replication stress. *CTRL* control, *UNT* untreated

Since DNA replication stress activates the ataxia telangiectasia mutated and Rad3-related protein (ATR)/Checkpoint kinase 1 (Chk1) response, we investigated whether these kinases may mediate APOBEC3 activity following hydroxyurea-induced DNA replication stress. MCF10A cells were first treated with ATR and ATM kinase inhibitors and APOBEC3 activity was assessed. Inhibition of ATR and to a lesser extent ATM led to a reduction of the hydroxyurea-induced APOBEC3 activation (Fig. 5a). Furthermore, treatment of MDA-MB-453 and BT474 cells with the novel specific Chk1 inhibitor CCT244747 [34] led to a reduction of basal APOBEC3 activity (Fig. 5b; Additional file 10: Figure S10a). Similarly, treatment of MCF10A cells with CCT244747 led to a reduction in hydroxyurea-induced APOBEC3B protein and activity (Fig. 5c, d). In addition, MCF10A cells were depleted of *ATR* or *CHEK1* by siRNA and subsequently APOBEC3 activity was measured under basal conditions and in response to hydroxyurea treatment. In the control siRNA transfected cells, hydroxyurea treatment caused robust APOBEC3 activation; in contrast, this response was reduced following depletion of *ATR* or *CHEK1* (Additional file 10: Figure S10b, c). Consistent with these findings, Chk1 inhibition using an alternative Chk1 inhibitor, UCN01, also prevented the hydroxyurea-dependent increase in *APOBEC3B* transcription (Additional file 10: Figure S10d). Interestingly, *APOBEC3B* expression correlated with sensitivity to the Chk1 inhibitor CCT244747 (Additional file 10: Figure S10e). There was no correlation between drug-induced cytotoxicity or changes in cell cycle distribution and APOBEC3 levels following treatment with the ATR, ATM or Chk1 inhibitors (Additional file 9: Figure S9b; Additional file 10: Figure S10f–h).

### Elevated replication stress-associated DNA damage in HER2-positive breast carcinomas

Since we observed that most HER2+ cell lines exhibited high levels of *APOBEC3B* mRNA expression and replication stress-associated G1 nuclear bodies, we next investigated whether the same characteristics were observed in vivo. We performed immunohistochemical analysis on sections from formalin-fixed, paraffin-embedded material from a clinical cohort of breast carcinomas and adjacent normal breast tissue with antibodies against γH2AX and phosphorylated RPA (RPA-P), markers of DNA damage and ATR signalling, respectively (Additional file 11: Figure S11a). We also tested commercially available APOBEC3B antibodies for suitability in immunohistochemistry but none were sufficient to reliably and specifically detect nuclear APOBEC3B. Compared with luminal breast tumours, the HER2+ tumours exhibited significantly higher proportion of samples with more than 10 % of cells staining positive for γH2AX (*p* = 3.3e-06, Fishers exact test; Additional file 11: Figure S11b). HER2+ tumours also exhibited more RPA-P than normal breast tissue (Additional file 11: Figure S11c). In addition, all but one case among the tumours exhibiting a high proportion of cancer cells positive for the γH2AX marker showed concomitant high levels of RPA-P, suggesting that replication stress contributes to the DNA damage observed in the HER2+ tumours. These results strengthen our conclusion that HER2+ tumours, which are associated with the highest mutational load of APOBEC3 mutations, exhibit high levels of replication stress-associated DNA damage.

## Discussion

The APOBEC3 mutational signature has been found in a range of different cancer types and is one of the most frequently occurring mutational signatures in the cancer genome [6]. It is not clear how APOBEC3 influences tumour evolution but it is hypothesized to increase the fitness of cancer cells by providing the beneficial gain of oncogene or loss of tumour suppressor gene function [23] and is associated with the progressive acquisition of subclonal mutations later in tumour evolution in some cancer types [13]. In breast cancer, the HER2+ subtype is especially affected by T*C*W mutations, indicating that APOBEC3 has been active during the progression of this disease [9]. It was found that HER2-amplified [9] and HER2-overexpressing tumours [35] harbour a high SCNA load, suggesting that HER2 might underlie the formation of SCNAs and APOBEC3 activity.

In addition to the activity of APOBEC3, the localization of APOBEC3 family members is also regulated. The subcellular localization of APOBEC3 family members has been evaluated using a series of green fluorescent protein fusion constructs [36–38]. In contrast to APOBEC3B, which is predominantly nuclear, APOBEC3G is cytoplasmic under steady state conditions [36–38]. However, in lymphoma cells APOBEC3G has been shown to enter the nuclear compartment as part of a DNA damage response and promote DNA repair by activating the ataxia telangiectasia mutated (ATM) DNA damage checkpoint kinase [39]. These data suggest potential roles for multiple APOBEC3 members in response to DNA damage and replication stress.

In this study, we first investigated the correlation between basal APOBEC3 activity and HER2 status. We identified a trend towards HER2-enriched breast cancer-derived cell lines having high basal APOBEC3 activity. The high levels of APOBEC3 activity in the HER2+ subtype indicates that an increased gene dosage of *ERBB2* may drive APOBEC3 in breast cancer, corroborating earlier observations by Roberts and colleagues [9].

Nevertheless, apart from *ERBB2* amplifications we expect other oncogenes to drive APOBEC3 activity by virtue of their impact on DNA replication stress. Oncogene activation can induce replication stress in several different ways, including interfering with the timing and number of origins firing [30, 32]. Furthermore, oncoproteins such as cyclin E may also induce replication stress via inactivation of retinoblastoma protein and enhanced S phase entry [2, 3]. Overexpression of RAS causes an initial hyperproliferation accelerating G1/S phase transition and we identified that PI3K and MAPK signalling contribute to APOBEC3 activity in multiple different breast cancer cell lines. This is consistent with previous data reporting the ERK signalling pathway is involved in the regulation of APOBEC3G in human T lymphocytes [40]. We also investigated APOBEC3 activity before and after RAS hyperactivation in MCF10A-ER:HRAS cells. RAS hyperactivation modestly induced APOBEC3 activity in these cells, suggesting that it could be insufficient to robustly activate APOBEC3 in tumours on its own. Since inhibition of both PI3K and MAPK pathways was shown to reduce APOBEC3 activity, one possibility is that both signalling pathways are required to activate APOBEC3 levels. Interestingly, we identified that mutations in *PTEN* and *NF1* were also associated with APOBEC3 mutation enrichment in the different breast cancer subtypes, further implicating PI3K and MAPK signalling in co-regulating APOBEC3 mutagenesis in breast cancer. Recent reports also show NFkB signalling plays a role in the regulation of APOBEC3B [41] and AID [42]. It is likely, therefore, that additional pathways drive APOBEC3 activity in cancer.

In addition to genetic and signalling factors, we identified that specific drugs can activate APOBEC3 activity. We found that exposure of cells to hydroxyurea, aphidicolin, gemcitabine and camptothecin resulted in enhanced APOBEC3 activity. These drugs were modestly associated with S phase enrichment. A similar observation was made in yeast after exposure to hydroxyurea [43]. These treatments also resulted in the highest levels of RPA-P, indicative of increased exposure of ssDNA. Hydroxyurea and gemcitabine can both inhibit ribonucleotide reductase, aphidicolin inhibits DNA polymerase alpha whereas camptothecin is a topoisomerase I inhibitor. As the extent of RPA-P achieved was highest with these drugs, we speculate that the differentiation between these drugs and the other tested DNA damaging agents is their ability to induce sufficient ssDNA provoking downstream DNA replication stress detection in order to surpass the threshold of ATR signalling required for APOBEC3 activation. Our findings might have clinical implications as we show that several clinically used cytotoxic drugs may induce APOBEC3 activity. Future studies should test the ability of other cytotoxic drugs to induce APOBEC3 activity and investigate whether relapse samples contain more therapy-induced APOBEC3 mutations.

Besides inducing APOBEC3 activity, we were also able to diminish APOBEC3 activation through supplementing growth media with exogenous nucleosides, which is known to rescue DNA replication stress and genomic instability [26, 27]. In addition we show that APOBEC3 activation is coordinated by a transcriptional response downstream of the key signalling cascades governed by ATR/ATM and this signalling can be rescued by exogenous nucleoside supplementation. These data implicate a role for replication-associated damage in triggering the transcriptional response to APOBEC3 activation. Interestingly, we found that two different Chk1 inhibitors potently inhibited APOBEC3 activity. Since ATR/Chk1 inhibition would lead to a failure to signal the presence of exposed ssDNA, these data are consistent with a role for this signalling cascade in mediating the DNA replication stress-invoked pathway following ssDNA exposure to APOBEC3 mRNA transcriptional activation. These findings are of potential clinical relevance as an analogue of CCT244747 will soon be tested in a phase 1 clinical trial and biomarkers of response to therapy are actively under investigation [44]. Additionally, we found a trend between *APOBEC3B* expression and sensitivity to CCT244747 (*p* value = 0.058, Spearman rank correlation test; Additional file 10: Figure S10e), which further strengthens the potential clinical relevance of our findings. We speculate that high levels of replication stress results in the activation of Chk1 and the subsequent induction of APOBEC3 activity. These cells exhibit high levels of DNA replication stress and are, therefore, more likely to be dependent on Chk1 signalling for repair [45], which may explain why we observe that cells with high APOBEC3 activity appear more sensitive to Chk1 inhibition. We speculate that more direct gene expression biomarkers, apart from *APOBEC3B*, could potentially be of use to predict response to CCT244747. Although more research is needed, we speculate that whereas replication stress-induced *APOBEC3B* regulation could contribute to a portion of APOBEC3-mediated mutagenesis, *APOBEC3G* upregulation could enhance DNA repair, as has been previously postulated in lymphoma cells [39]. Our *in vitro* data are supported by our observation that replication stress-associated DNA damage was significantly associated with the HER2+ subtype, suggesting that replication stress could potentially drive APOBEC3 activation in HER2+ breast carcinomas.

We recently showed in ER-negative breast cancer that there is a modest increase in the APOBEC3 mutation signature in later, subclonal mutations, implicating this process in the exacerbation of intratumour heterogeneity in ER-negative breast cancer [13]. Our current data suggests that this late activation could be a response to altered levels of replication stress (Fig. 5e). DNA replication stress and its induction through mitogenic signalling could be a particularly potent driver of genomic instability, inducing both chromosomal instability and APOBEC3 activation (Fig. 5e).

## Conclusions

These findings illustrate that DNA replication stress appears to be a particularly potent driver of genomic instability in cancer. On the one hand, DNA replication stress has been demonstrated to induce both structural and numerical chromosomal aberrations generating chromosomal instability [27]. In addition, we now propose that replication stress is able to induce single nucleotide-level mutagenesis mediated via *APOBEC3B* induction. These dual roles for DNA replication stress in mediating genomic instability could link the high level of SCNAs and single nucleotide diversity caused by APOBEC3 activity that are both observed in HER2+ tumours.

We have found that oncogenic signalling, cytotoxic drugs and genetic modulators of replication stress are all able to modulate APOBEC3 activity. These findings implicate the ability of therapeutics that either attenuate oncogenic signalling or exacerbate DNA replication stress to alter cancer’s mutagenic landscape and evolutionary potential.

## Methods

### Cell lines

SKBR3, MCF7, HCC1419, HCC1569, HCC1954, T47D, BT474, MDA-MB-231, MDA-MB-361, MDA-MB-453, ZR75.1, MDA-MB-134, CAL51, HCC1428 cell lines were obtained from The Francis Crick Institute, Cell services. All cells were grown in RPMI 1640 media (ThermoFisher Scientific), supplemented with 10 % foetal bovine srum and 1/10,000 units of penicillin-streptomycin (Sigma-Aldrich) and with L-glutamine (ThermoFisher Scientific). MCF10A cells were a kind gift from Almut Schulze. MCF10A-ER:HRAS were a kind gift from Julian Downward. MCF10A and MCF10A-ER:HRAS cells were grown in DMEMF12 supplemented with 5 % horse serum, 10 μg/ml insulin, 5 μg/ml hydrocortisone, 20 ng/ml epidermal growth factor, 100 ng/ml cholera toxin and 1/10000 units of penicillin-streptomycin. All cell lines used in this study were maintained at 37 °C in 5 % CO_2._

### Treatments

Where indicated, cells were treated with 2 mM hydroxyurea, 2.5 μm aphidicolin, 10 μm etoposide, 10 μm 5-fluorouracil, 1 μM cisplatin, 1 μM gemcitabine, 0.001 % methyl methanesulfonate, 100 nM doxorubicin, 3 μM camptothecin, 300 μM nucleosides, 10 nM afatinib, 30 nM lapatinib, 150 nM rapamycin, 15 μm MEK inhibitor U0126, 150 nM AKT inhibitor MK2206, 7.5 μm PI3K inhibitor LY294002, 1 μm ATR kinase inhibitor VE821 (AdooQ), CCT244747 (a kind gift from Prof. Ian Collins, ICR, London), 10 μm ATM kinase inhibitor KU55933 (Merck, Millipore), 100 nM UCN01 Chk1/PKCβ inhibitor (Merck, Millipore), 12.5–300 μM EmbryoMax Nucleosides (Millipore).

### RNA interference

All siRNA (Dharmacon, GE Healthcare) transfections were performed at 40 nM final concentrations by reverse transfection with Lipofectamine® RNAiMax (Thermo Fisher Scientific): *ATR* (L-003202), *CHEK1* (L-003255), *ERBB2* (LU-003126), *RRM1* (LU-004270), *RRM2* (LU-010379), *RRM2B* (LU-010575), *PTEN* (J-003023), *APOBEC3B* (J-017322). Non-targeting (NT) control siRNA was used as control in all experiments.

### RNA extraction and reverse transcription PCR

RNA was extracted using a Qiagen RNeasy kit and then reverse transcribed to cDNA using an AffinityScript cDNA synthesis kit (Agilent Technologies) according to the manufacturers’ instructions. Quantitative PCR was performed with triplicates in 96-well plate format on the StepOnePlus Real-Time PCR system (ThermoFisher Scientific) using pre-designed TaqMan® probes for *APOBEC3B* (Hs00358981_m1) and *APOBEC3G* (Hs00222415_m1) and *ATR* (Hs00992123_m1). *RRM1*, *RRM2*, *RRM2B*, *PTEN* and *CHEK1* quantitative PCR was performed using QuantiTect SYBR Green PCR kits. mRNA expression levels were quantified using the comparative Ct method, normalized to DNA topoisomerase I (Hs00243257_m1).

### Gel-based deamination assay using oligonucleotide probe

We seeded 200,000 cells per well in six-well plates. Cells were allowed to adhere for 24 h, after which they were treated with cytotoxic drugs or siRNAs for up to 72 h. Cells were subsequently isolated and lysed in HED buffer (25 mM HEPES, 5 mM EDTA, 10 % glycerol, 1 mM DTT (added fresh) and protease inhibitor (added fresh)). The protein concentrations were equalized and deamination reactions were performed at 37 °C for 3 h using the APOBEC3 probe 1 (5′-fluorescein-ATTATTATTATTATTCCCAATTATTTATTTATTTATTTATTT) [46] or probe 2 (5′-ATTATTATTATTCGAATGGATTTATTTATTTATTTATTTATTT-fluorescein-3′) in a 10× UDG reaction buffer consisting of 1.25 μL RNaseA (0.125 mg/mL), 1 μL probe (0.2 pmol/μL), 16.5 μL cleared lysate and uracil DNA glycosylase (UDG; New England Biolabs, 1.25 units). We added 100 mM NaOH and the sample was then incubated at 95 °C for 30 minutes to cleave the abasic sites followed by addition of formamide-based gel sample buffer. The reaction product was run on a 15 % urea-TBE gel that was imaged and quantified on an ImageQuant LAS 4000. Probe 1 was used in all experiments unless stated otherwise.

### Cytotoxicity assay

The cytotoxicity of CCT244747 was determined using a sulforhodamine-based growth delay assay. Cells were plated at appropriate densities into 96-well plates and allowed to attach for 36 h. Drug treatment was from 4 to 10 days to allow drug contact for at least two doubling times followed by sulforhodamine B staining and 50 % growth inhibition (GI50) determination. GI50 values were determined using Graph Pad Prism 6 software and Spearman rank correlations were performed between GI50 values against *APOBEC3B* mRNA expression and APOBEC3 activity.

### Flow cytometry analysis

Cells were washed in PBS and resuspended in PBS/0.1 % bovine serum albumin (BSA) and DNA was stained with propidium iodide. Samples were analyzed on a BD LSRFortessa X-20 cytometer (BD Biosciences) and processed in FlowJo.

### Cell viability assay

Treatment-induced cytotoxicity was determined using the CellTiter-Glo luminescent cell viability kit (Promega) in accordance with the manufacturer’s instructions.

### Protein extraction and western blotting

Total cell lysates were generated as described previously [47, 48]. Following SDS-PAGE, blots were probed with indicated antibodies diluted in 5 % milk or BSA in Tris-buffered saline. Antibodies: HER2 (Cell Signaling #2248), pSer473 AKT (Cell Signaling #4060), total AKT (Cell Signaling #2920), rabbit anti-APOBEC3B monoclonal antibody 5210-87-13 [41], HRP-conjugated anti-β-GAPDH antibody (Abcam ab9482) and HRP-conjugated goat anti-mouse/rabbit immunoglobulins (Dako). Immobilon Western Chemiluminescent HRP Substrate (Millipore) was used for detection.

### Immunofluorescence

Cells were treated and fixed as described previously [47]. Cells were stained with the indicated antibodies: 53BP1 (sc22760 Santa Cruz), cyclin A (in house), Ser139-γH2AX (Millipore 05636), RPA32/RPA2 (phosphoS4 + S8, Abcam ab87277). Anti-mouse and anti-rabbit IgG (H + L) Alexa Fluor 488, 594 and 647 secondary antibodies were used at 1:500 dilution (ThermoFisher Scientific).

### Archival tumour samples and immunohistochemistry

Formalin fixed, paraffin-embedded specimens of normal breast tissue adjacent to tumour (*n* = 37) and breast carcinoma tissues (*n* = 120) from the tissue archive of the Danish Cancer Society Research Center in Copenhagen were examined. All tissue samples were collected from patients who underwent a mastectomy between 2003 and 2012. None of the patients had previously undergone surgery involving the breast and they did not receive preoperative treatment. Tumour subtype scoring of luminal (*n* = 66; luminal A + B) and HER2 (*n* = 54) was performed based on estrogen receptor (ER), progesterone receptor (PGR), human epidermal receptor-2 (HER2), and an average Ki67 expression in accordance with St. Gallen International Breast Cancer Guidelines [49]. A HER2 gene copy score of two was evaluated by DNA FISH where a value <2.2 was considered negative and ≥2.2 was considered positive. For immunohistochemical staining and analysis, the paraffin tissue sections (4 μm) were deparaffinized in xylene and rehydrated in a graded series of ethanol-aqueous solutions. Antigen retrieval was carried out in 10 mM citrate buffer (pH 6.0) by heating the slides for 20 minutes in a microwave oven. Endogenous peroxidase activity was blocked by incubating the sections in 3 % hydrogen peroxide in Tris-buffered saline for 10 minutes. The primary antibodies were incubated overnight. The following primary antibodies were used: mouse monoclonal anti-phospho-histone H2AX (Ser139, Millipore; diluted 1/2000) and rabbit polyclonal anti-phospho-RPA32 (Thr21, Abcam, diluted 1/250). Normal non-immune serum served as a negative control. The primary antibodies were incubated overnight, followed by detection using the Vectastain Elite kit according to the manufacturer’s instructions (Vector Laboratories, Burlingame, CA, USA) and nickel sulphate enhancement without nuclear counterstaining, as described [2]. Immunostaining patterns on each slide were scored by an experienced oncopathologist based on the fraction of positive nuclear staining signals (counting a minimum of 300 epithelium or tumour cell nuclei per slide) and the threshold for scoring the categories of positivity was as follows: <2 % positive nuclei; 2–10 % positive nuclei; and >10 % positive nuclei.

### Breast cancer subtype classification and APOBEC3 mutation pattern detection

Data on breast cancer tumours (*n* = 755) were obtained from TCGA Research Network (http://cancergenome.nih.gov/). Tumours were previously divided into subtypes HER2, basal, luminal A, luminal B and normal based on the PAM50 method [21]. Primary data, including SNP6 copy number profiles, mutation calls and APOBEC3 enrichment values, were obtained from TCGA data version 2016_01_28. The APOBEC3 enrichment as a numeric value for the strength of APOBEC3 mutagenesis is calculated similarly to [9] as:

$$ \mathrm{E} = \kern0.5em \frac{\mathrm{mutationsTCW}\ \mathrm{X}\ \mathrm{contextC}}{\mathrm{mutationsC}\ \mathrm{X}\ \mathrm{contextTCW}} $$

The parameter ‘mutationsTCW’ displays the number of mutated cytosines in a TCW motif or mutated guanines in a WGA motif. ‘MutationsC’ represents the total number of mutated cytosines (or guanines), ‘contextTCW’ represents the total number of TCW (or WGA) motifs and context, the total number of cytosines (or guanines) in a specific region centred within 20 nucleotides before and 20 nucleotides after the mutated cytosines (or guanines).

Tumour samples that were significantly enriched for APOBEC3 signature mutations (Benjamini–Hochberg corrected *p* value <0.05) and fold enrichments >2 were classified as ‘APOBEC high’, and the rest as ‘APOBEC low’. To test whether genes were associated with APOBEC3 enrichment, a permutation test was carried out with 100,000 permutations, randomly shuffling the labels between ‘APOBEC high’ and ‘APOBEC low’ samples. The entire cohort was first tested together to determine which genes were significantly associated, following which the test was performed again for each gene within each subtype.

## Additional files

Additional file 1: Figure S1.
*APOBEC3B* expression and activity in breast cancer cell lines. **a** Boxplot showing number of SCNA breakpoints in APOBEC high versus APOBEC low samples. **b** APOBEC3 isoform expression in 15 breast cancer cell lines (gene expression data for *APOBEC3B* extracted from CCLE_Expression_Entrez_2012-10-18.res:Gene-centric RMA-normalized mRNA expression data). HER2+ cell lines (*red*), basal cell lines (*black*), luminal cell lines (*green*). SKBR3 cells have a null mutation for *APOBEC3B*. **c** *APOBEC3B* was depleted from MDA-MB-453, HCC1569 and BT474 cells by RNAi for 72 h, followed by lysis and APOBEC3 cytidine deamination assay. **d** MDA-MB-453, HCC1569 and BT474 cells were treated as in **c** followed by quantitative PCR for *APOBEC3B* to determine the extent of knockdown. **e** Eight cell lines were lysed and subjected to oligonucleotide-based cytidine deamination assay for APOBEC3 activity using probe 1. **f** Eight cell lines were lysed and subjected to oligonucleotide-based cytidine deamination assay for APOBEC3 activity using probe 2. **g** Spearman rank correlation between APOBEC3 activity and *APOPEC3B* mRNA expression in the panel of 15 cell lines used in Fig. 2 (r = 0.8, *p* = 0.0016). **h** Spearman rank correlation between the fraction of G1 nuclear bodies and the *APOPEC3B* mRNA expression level in the panel of 15 cell lines used in Fig. 2. **i** BT474 cells were treated with 300 μM nucleosides for 48 h. Cell viability was determined by CellTiter-Glo. **j** BT474 cells were treated with 300 μM nucleosides followed by analysis of cell cycle distribution by FACS. (TIF 35303 kb) Additional file 2: Figure S2. Induction of replication stress in cell lines using cytotoxic drugs **a** MCF10A cells, **b** MCF7 cells, **c** HCC1419 cells, **d** MDA-MB-134 cells and **e** SKBR3 cells were treated with the indicated drugs for 48 h prior to lysis and cytidine deamination assays for APOBEC3 activity. **f** MCF7 and CAL51 cells were treated with siRNAs targeting APOBEC3B for 72 h followed by lysis and cytidine deamination assay for APOBEC3 activity. **g** MCF7 and CAL51 cells were treated as in **f** followed by quantitative PCR to determine levels of APOBEC3 depletion. The quantitative PCR was normalized according to siNT control. (TIF 34618 kb) Additional file 3: Figure S3. Cell viability following treatment of breast cancer cell lines with cytotoxic drugs and nucleosides. **a** MCF10A cells, **b** MDA-MB-134 cells, **c** HCC1419 cells and **d** HCC1428 cells were incubated with the indicated drugs for 48 h, followed by cell viability determination by CellTiter-Glo. **e** MCF10A cells were pre-treated with or without 300 μM nucleosides for 24 h followed by 2 mM hydroxyurea treatment for an additional 48 h. Cell cycle distribution was determined by FACS analysis. (TIF 34064 kb) Additional file 4: Figure S4. Cell cycle distribution following treatment of breast cancer cell lines with cytotoxic drugs. **a** MCF10A cells were incubated with the indicated drugs for 48 h prior to harvesting and determination of cell cycle distribution by FACS analysis. **b** Histogram representing the results shown in **a** displaying the percentage of cells in each cell cycle phase in response to the different treatments. **c** HCC1419 cells were incubated with the indicated drugs for 48 h prior to harvesting and determination of cell cycle distribution by FACS analysis. **d** MDA-MB-134 cells were incubated with the indicated drugs for 48 h prior to harvesting and determination of cell cycle distribution by FACS analysis. (TIF 34520 kb) Additional file 5: Figure S5. Titration of cytotoxic drugs in breast cancer cell lines. **a** MCF10A cells were incubated with the indicated concentrations of hydroxyurea for the indicated times prior to lysis and cytidine deamination assays for APOBEC3 activity. **b** MCF10A cells were treated with the indicated of doses of hydroxyurea for 48 h prior to lysis and cytidine deamination assay for APOBEC3 activity. **c** MCF10A cells were treated as in **b** followed by lysis and probing western blots with the indicated antibodies. MCF10A cells were treated with the indicated concentrations of aphidicolin (**d**), gemcitabine (**e**) and cisplatin (**f**) for 48 h prior to lysis and cytidine deamination assays for APOBEC3 activity. (TIF 34567 kb) Additional file 6: Figure S6. Replication stress correlates with APOBEC3 activation. **a** MCF7 cells were treated with the indicated drugs for 48 h followed by fixation and immunofluorescence for Ser139 γH2AX and S4/8 RPA phosphorylation. *Red asterisks* indicate treatments inducing APOBEC3 activity levels and S4/8 RPA phosphorylation. Spearman rank correlations between **b** the extent of RPA phosphorylation and *APOBEC3B* mRNA expression, **c** the extent of RPA phosphorylation and *APOBEC3G* mRNA expression, **d** the extent of RPA phosphorylation and APOBEC3 activity (arbitrary units) from MCF10A cells treated in Fig. 3. **e** Quantitative PCR validation of knockdown of RRM subunits to accompany Fig. 3f. 72 h after siRNA transfection, MCF10A cells were lysed and mRNA isolated followed by cDNA synthesis. Quantitative PCR was performed to determine expression levels of *RRM1*, *RRM2* and *RRM2B*. (TIF 38074 kb) Additional file 7: Figure S7. Role of receptor tyrosine kinase signalling in APOBEC3 activation. **a** BT474 and MDA-MB-361 cells were treated with RNAi targeting *ERBB2*. After 72 h, cells were harvested, lysed and western blots were probed with the indicated antibodies to determine the extent of ERBB2 silencing. **b** BT474 cells were treated with 10 nM afatinib or 30 nM lapatinib for 24 h followed by lysis. Western blots were probed with the indicated antibodies. **c** HCC1419 cells were treated with 2 mM hydroxyurea in the presence or absence of 30 nM lapatinib. Following mRNA isolation and cDNA synthesis, *APOBEC3B* mRNA expression levels were determined by quantitative PCR. **d** HCC1419 cells were treated as in **c** and, following lysis, oligonucleotide-based cytidine deamination assays were performed for APOBEC3 activity using probe 1. **e** HCC1419 cells were treated as in **c** and, following lysis, APOBEC3 cytidine deamination assays were performed using probe 2. **f** HCC1419 cells were treated as in **c**. Cells were lysed and western blots were probed with the indicated antibodies. (TIF 34667 kb) Additional file 8: Figure S8. Role of MAPK and PI3K pathways in APOBEC3 activation. **a** BT474 cells were treated with 10 nM afatinib or 30 nM lapatinib for 24 h followed by cell viability determination by CellTiter-Glo. **b** BT474 cells were treated with the indicated drugs for 48 h followed by cell viability determination by CellTiter-Glo. **c** BT474 cells were treated as in **a** and **b** followed by analysis of cell cycle distribution by FACS. **d** MCF10A cells were treated with the indicated drugs as in Fig. 4g followed by RNA isolation, cDNA synthesis and quantitative PCR to determine *APOBEC3G* levels. **e** BT474 cells and **f** HCC1569 cells were treated with the indicated drugs for 48 h. Cells were lysed and oligonucleotide-based cytidine deamination assays were performed for APOBEC3 activity. (TIF 37363 kb) Additional file 9: Figure S9. Role of MAPK and PI3K pathways in hydroxyurea-induced APOBEC3 activation. **a** MCF10A cells were treated with the indicated drugs for 48 h followed by cell viability determination by CellTiter-Glo. **b** MCF7 cells were treated with the indicated drugs for 48 h followed by analysis of cell cycle distribution by FACS analysis. **c** MCF7 cells were transfected with *PTEN* siRNA. After 72 h the extent of knockdown was determined by quantitative PCR. **d** PTEN levels were depleted from MCF7 cells by RNAi. After 72 h cells were harvested and western blots were probed with the indicated antibodies. **e** MCF7 cells were transfected with *PTEN* siRNA or ubiquitin (*UBB*) control, followed by cell viability determination by CellTiter-Glo. **f** MCF7 cells were treated as in **c** followed by cell cycle distribution analysis by FACS. (TIF 35459 kb) Additional file 10: Figure S10. Role for DNA damage signalling in APOBEC3 activation. **a** BT474 cells were treated with the indicated doses of Chk1 inhibitor CCT244747 followed by APOBEC3 cytidine deamination assay. **b** *ATR* and *CHEK1* were depleted from MCF10A cells by RNAi. After 24-h transfection, cells were treated with hydroxyurea for a further 48 h prior to lysis and cytidine deamination assay for APOBEC3 actvity. **c** Validation of the extent of silencing of *ATR* and *CHEK1* in MCF10A cells. Cells were treated with the indicated siRNAs for 72 h followed by mRNA isolation, cDNA synthesis and quantitative PCR for *ATR* and *CHEK1* mRNA expression levels. **d** MCF10A cells were treated with hydroxyurea in the presence or absence of Chk1 inhibitor UCN-01 for 48 h prior to mRNA isolation, cDNA synthesis and quantitative PCR for *APOBEC3B* levels. **e** Eight breast cancer cell lines were treated with ten doses of CCT244747 for two population doublings followed by sulforhodamine B staining and GI50 determination. **f** MCF10A cells were treated with the indicated drugs for 48 h followed by cell viability determination using CellTiter-Glo. **g** BT474 cells were treated with 9 μM CCT244747 followed by analysis of cell cycle distribution by FACS. **h** MCF10A cells were treated with the indicated drugs for 48 h followed by analysis of cell cycle distribution by FACS. (TIF 34524 kb) Additional file 11: Figure S11. The HER2+ breast cancer subtype exhibits high levels of γH2AX and RPA-P staining. **a** Examples of immunohistochemical staining of normal breast and HER2+ tumour samples with Ser139 γH2AX and RPA-P. **b** The percentages of cases scoring <2 %, 2–10 % and >10 % or more positive for Ser139 γH2AX in each subtype. *P* = 3.314e-06, Fishers exact test, comparing luminal versus HER2+ subtype exhibiting more than 10 % of cells staining positive for Ser139 γH2AX. **c** The percentages of cases scoring <2 %, 2–10 % and >10 % or more positive for RPA-P in each subtype. (TIF 34565 kb)

## Abbreviations

APOBEC3: Apolipoprotein B mRNA editing enzyme, catalytic polypeptide-like 3
ATM: Ataxia telangiectasia mutated
BSA: Bovine serum albumin
CCLE: Cancer Cell Line Encyclopedia
DSB: double strand break
ER: Estrogen receptor
FDR: False discovery rate
GI50: 50 % growth inhibition
HER2: Human epidermal receptor-2
MAPK: Mitogen-activated protein kinase
mTOR: Mammalian target of rapamycin
PI3K: Phosphatidylinositol 3-kinase
RNAi: RNA interference
RPA: Replication protein A
RPA-P: Phosphorylated RPA
SCNA: Somatic copy number aberration
siRNA: Small interfering RNA
ssDNA: Single-stranded DNA
TCGA: The Cancer Genome Atlas
