# Supplementary figures and images for: DNA replication stress mediates APOBEC3 family mutagenesis in breast cancer
Source: Genome Biol. 2016 Sep 15;17:185. doi: 10.1186/s13059-016-1042-9 (PMC5025597; doi:10.1186/s13059-016-1042-9)

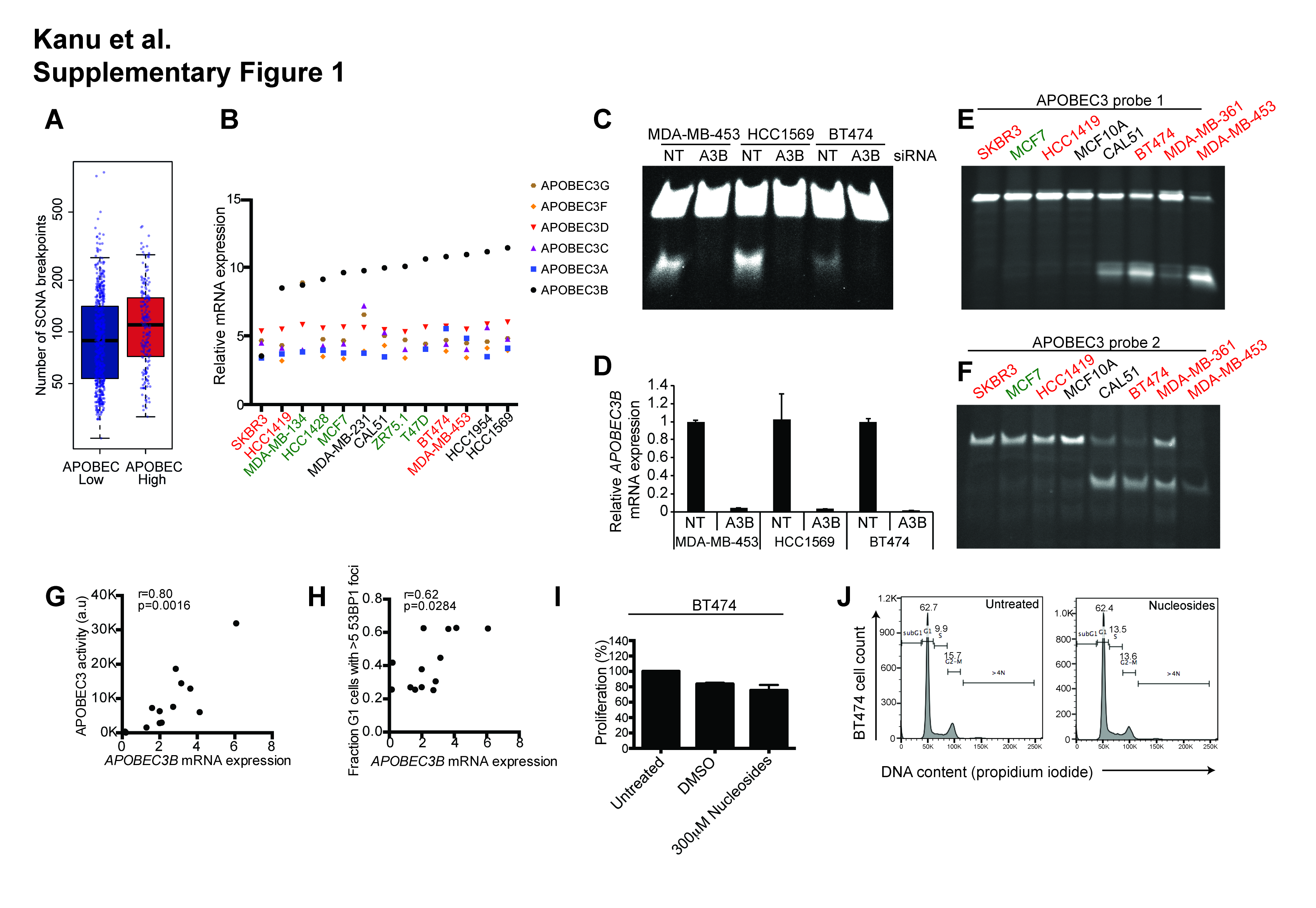

Supplement: Additional file 1: Figure S1. — APOBEC3B expression and activity in breast cancer cell lines. a Boxplot showing number of SCNA breakpoints in APOBEC high versus APOBEC low samples. b APOBEC3 isoform expression in 15 breast cancer cell lines (gene expression data for APOBEC3B extracted from CCLE_Expression_Entrez_2012-10-18.res:Gene-centric RMA-normalized mRNA expression data). HER2+ cell lines (red), basal cell lines (black), luminal cell lines (green). SKBR3 cells have a null mutation for APOBEC3B. c APOBEC3B was depleted from MDA-MB-453, HCC1569 and BT474 cells by RNAi for 72 h, followed by lysis and APOBEC3 cytidine deamination assay. d MDA-MB-453, HCC1569 and BT474 cells were treated as in c followed by quantitative PCR for APOBEC3B to determine the extent of knockdown. e Eight cell lines were lysed and subjected to oligonucleotide-based cytidine deamination assay for APOBEC3 activity using probe 1. f Eight cell lines were lysed and subjected to oligonucleotide-based cytidine deamination assay for APOBEC3 activity using probe 2. g Spearman rank correlation between APOBEC3 activity and APOPEC3B mRNA expression in the panel of 15 cell lines used in Fig. 2 (r = 0.8, p = 0.0016). h Spearman rank correlation between the fraction of G1 nuclear bodies and the APOPEC3B mRNA expression level in the panel of 15 cell lines used in Fig. 2. i BT474 cells were treated with 300 μM nucleosides for 48 h. Cell viability was determined by CellTiter-Glo. j BT474 cells were treated with 300 μM nucleosides followed by analysis of cell cycle distribution by FACS. (TIF 35303 kb) [file 13059_2016_1042_MOESM1_ESM.tif]

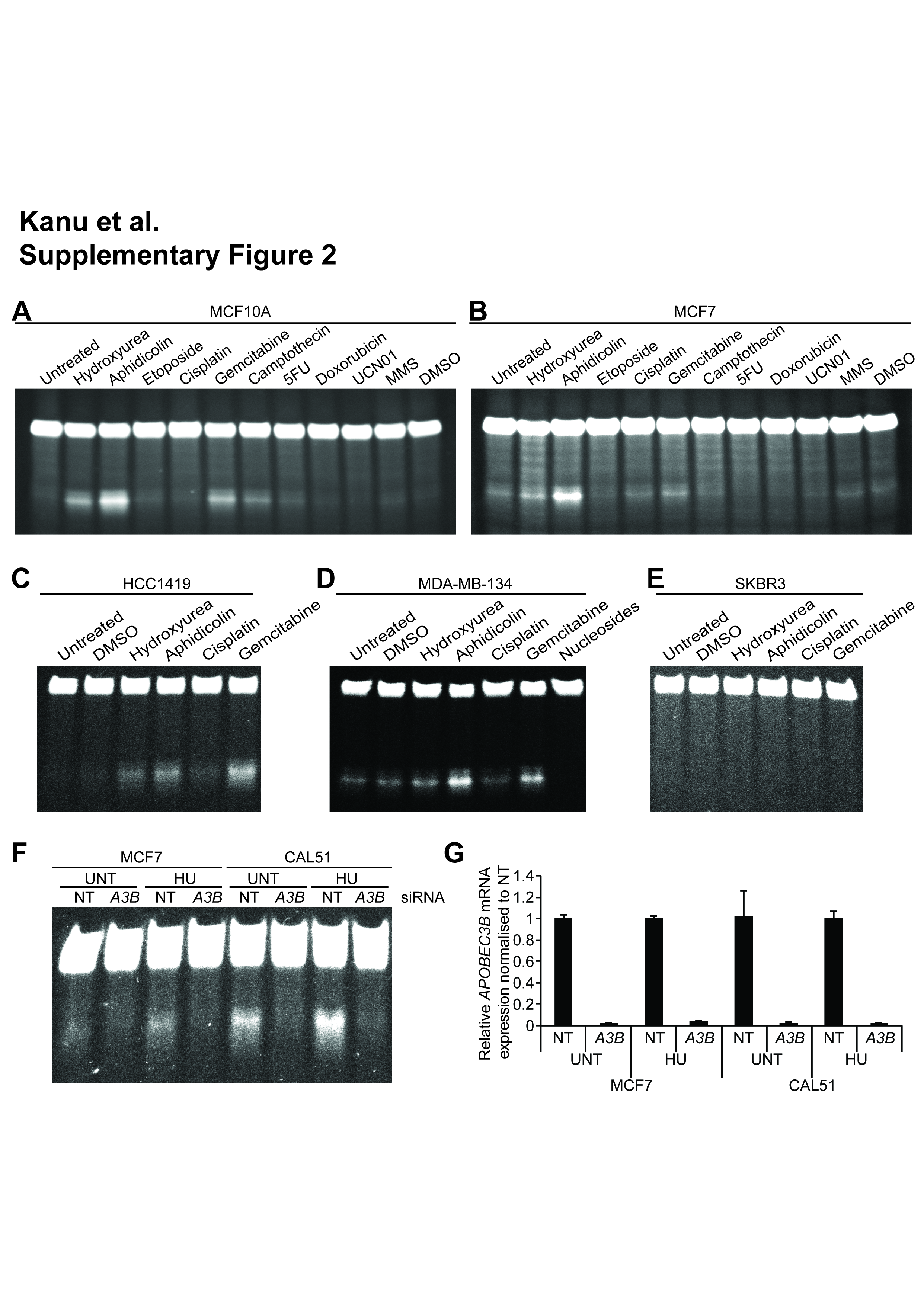

Supplement: Additional file 2: Figure S2. — Induction of replication stress in cell lines using cytotoxic drugs a MCF10A cells, b MCF7 cells, c HCC1419 cells, d MDA-MB-134 cells and e SKBR3 cells were treated with the indicated drugs for 48 h prior to lysis and cytidine deamination assays for APOBEC3 activity. f MCF7 and CAL51 cells were treated with siRNAs targeting APOBEC3B for 72 h followed by lysis and cytidine deamination assay for APOBEC3 activity. g MCF7 and CAL51 cells were treated as in f followed by quantitative PCR to determine levels of APOBEC3 depletion. The quantitative PCR was normalized according to siNT control. (TIF 34618 kb) [file 13059_2016_1042_MOESM2_ESM.tif]

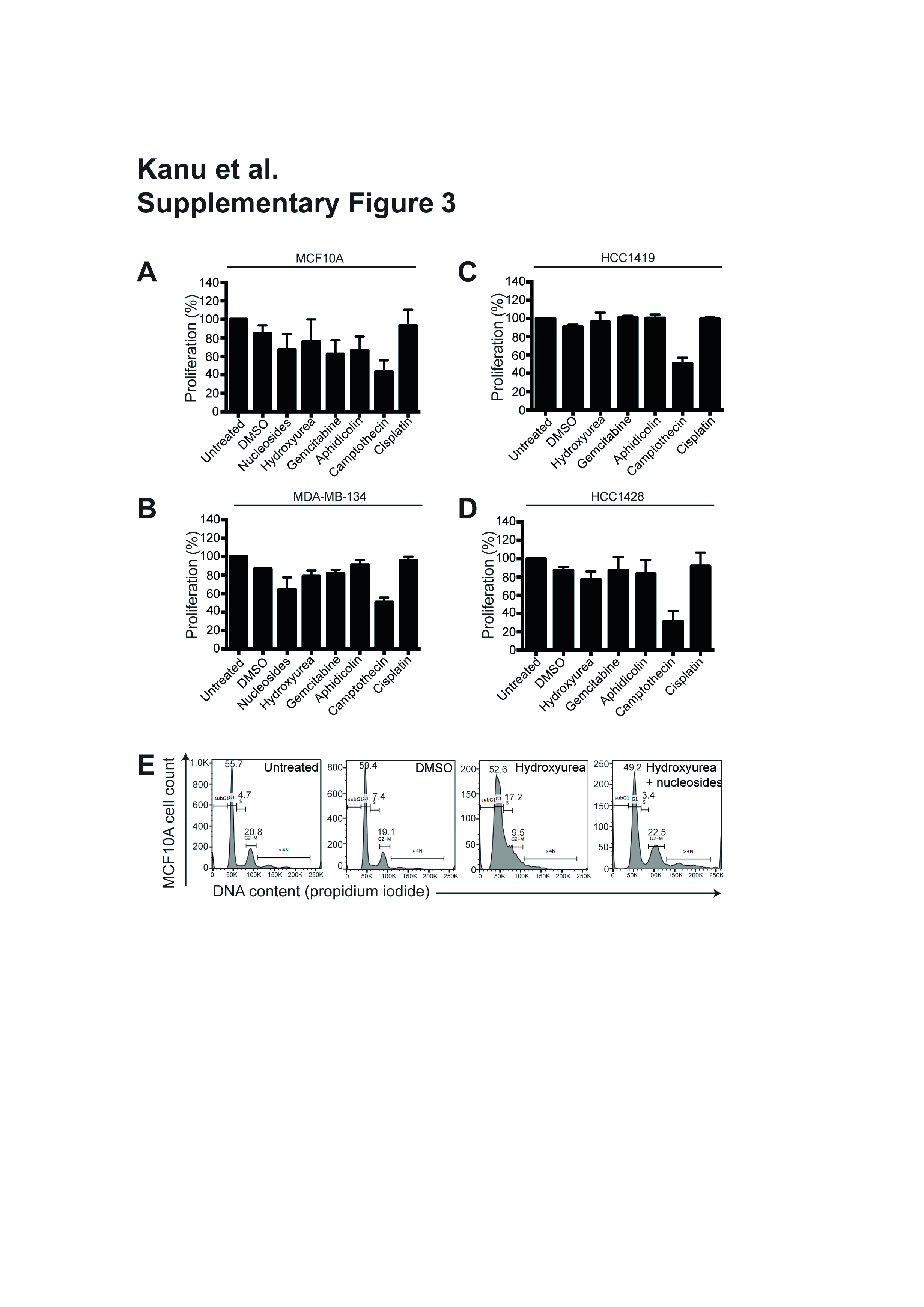

Supplement: Additional file 3: Figure S3. — Cell viability following treatment of breast cancer cell lines with cytotoxic drugs and nucleosides. a MCF10A cells, b MDA-MB-134 cells, c HCC1419 cells and d HCC1428 cells were incubated with the indicated drugs for 48 h, followed by cell viability determination by CellTiter-Glo. e MCF10A cells were pre-treated with or without 300 μM nucleosides for 24 h followed by 2 mM hydroxyurea treatment for an additional 48 h. Cell cycle distribution was determined by FACS analysis. (TIF 34064 kb) [file 13059_2016_1042_MOESM3_ESM.tif]

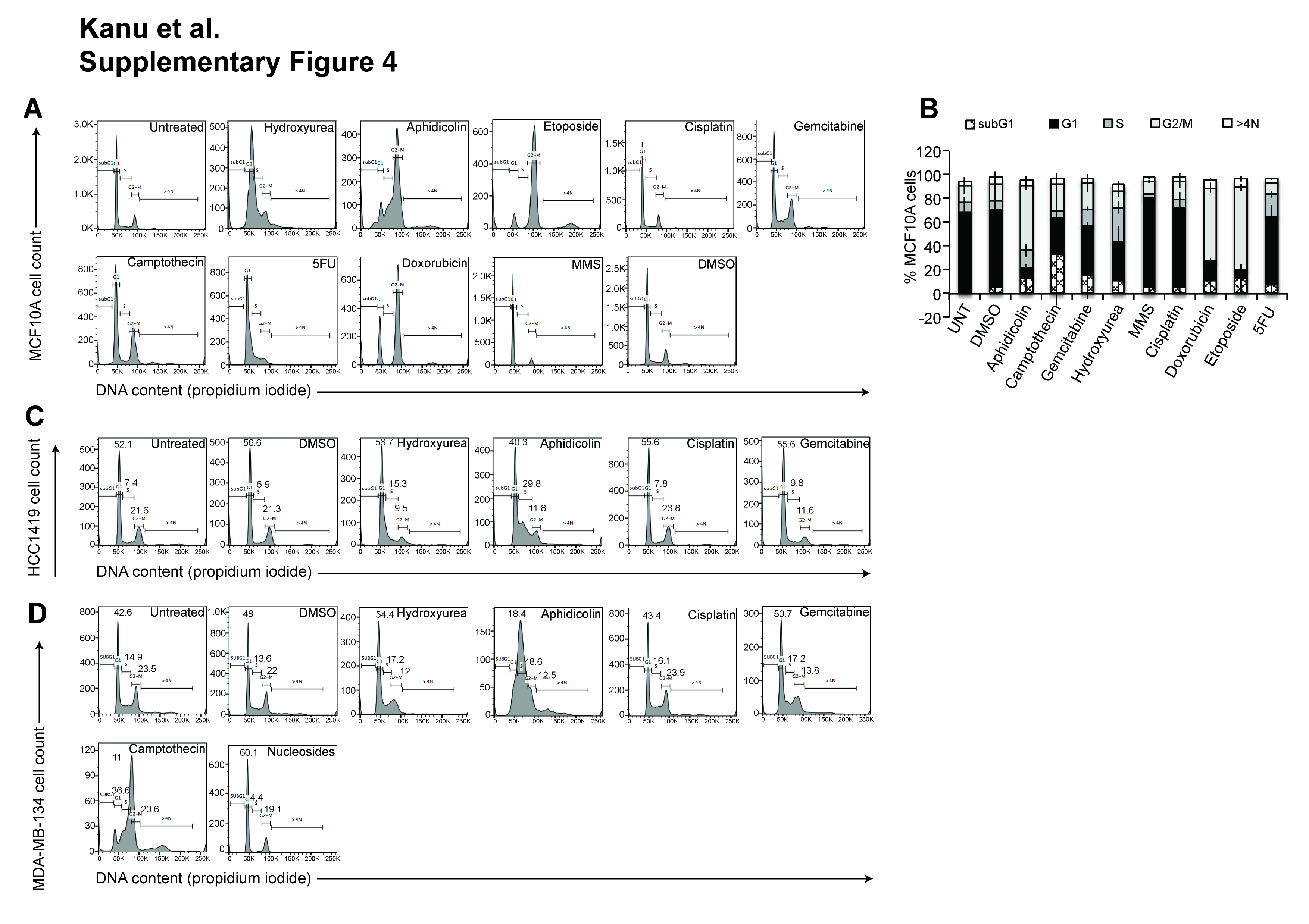

Supplement: Additional file 4: Figure S4. — Cell cycle distribution following treatment of breast cancer cell lines with cytotoxic drugs. a MCF10A cells were incubated with the indicated drugs for 48 h prior to harvesting and determination of cell cycle distribution by FACS analysis. b Histogram representing the results shown in a displaying the percentage of cells in each cell cycle phase in response to the different treatments. c HCC1419 cells were incubated with the indicated drugs for 48 h prior to harvesting and determination of cell cycle distribution by FACS analysis. d MDA-MB-134 cells were incubated with the indicated drugs for 48 h prior to harvesting and determination of cell cycle distribution by FACS analysis. (TIF 34520 kb) [file 13059_2016_1042_MOESM4_ESM.tif]

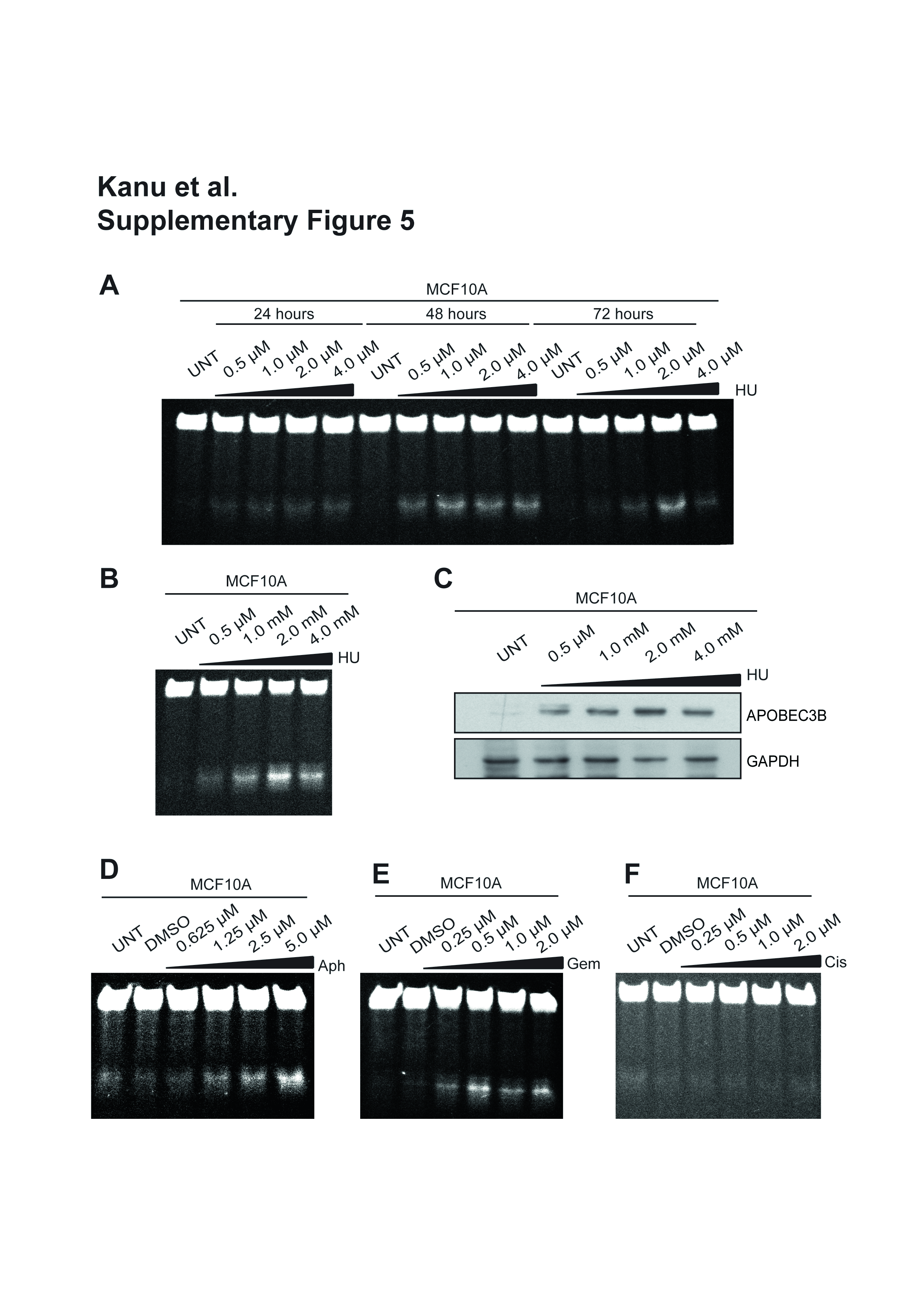

Supplement: Additional file 5: Figure S5. — Titration of cytotoxic drugs in breast cancer cell lines. a MCF10A cells were incubated with the indicated concentrations of hydroxyurea for the indicated times prior to lysis and cytidine deamination assays for APOBEC3 activity. b MCF10A cells were treated with the indicated of doses of hydroxyurea for 48 h prior to lysis and cytidine deamination assay for APOBEC3 activity. c MCF10A cells were treated as in b followed by lysis and probing western blots with the indicated antibodies. MCF10A cells were treated with the indicated concentrations of aphidicolin (d), gemcitabine (e) and cisplatin (f) for 48 h prior to lysis and cytidine deamination assays for APOBEC3 activity. (TIF 34567 kb) [file 13059_2016_1042_MOESM5_ESM.tif]

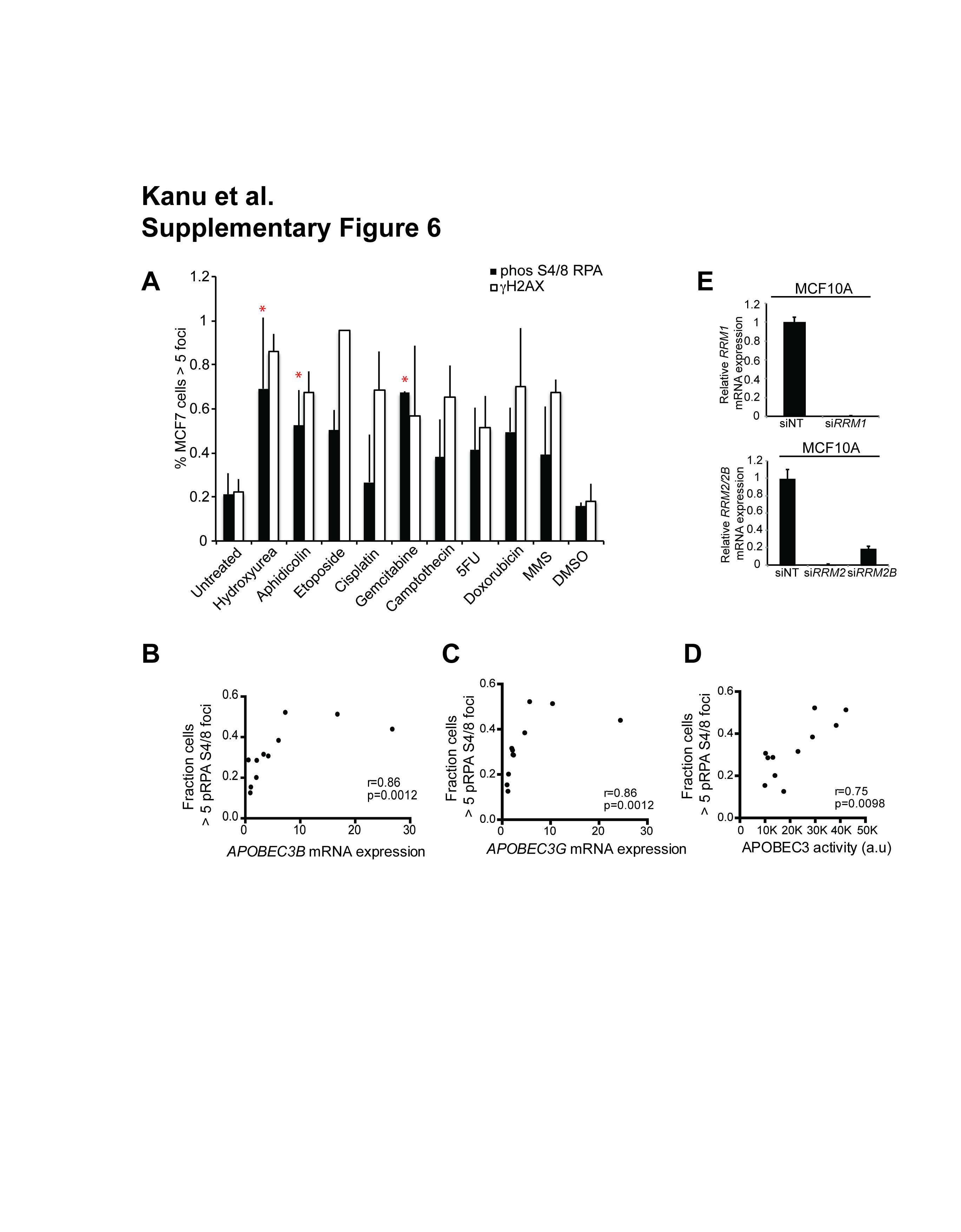

Supplement: Additional file 6: Figure S6. — Replication stress correlates with APOBEC3 activation. a MCF7 cells were treated with the indicated drugs for 48 h followed by fixation and immunofluorescence for Ser139 γH2AX and S4/8 RPA phosphorylation. Red asterisks indicate treatments inducing APOBEC3 activity levels and S4/8 RPA phosphorylation. Spearman rank correlations between b the extent of RPA phosphorylation and APOBEC3B mRNA expression, c the extent of RPA phosphorylation and APOBEC3G mRNA expression, d the extent of RPA phosphorylation and APOBEC3 activity (arbitrary units) from MCF10A cells treated in Fig. 3. e Quantitative PCR validation of knockdown of RRM subunits to accompany Fig. 3f. 72 h after siRNA transfection, MCF10A cells were lysed and mRNA isolated followed by cDNA synthesis. Quantitative PCR was performed to determine expression levels of RRM1, RRM2 and RRM2B. (TIF 38074 kb) [file 13059_2016_1042_MOESM6_ESM.tif]

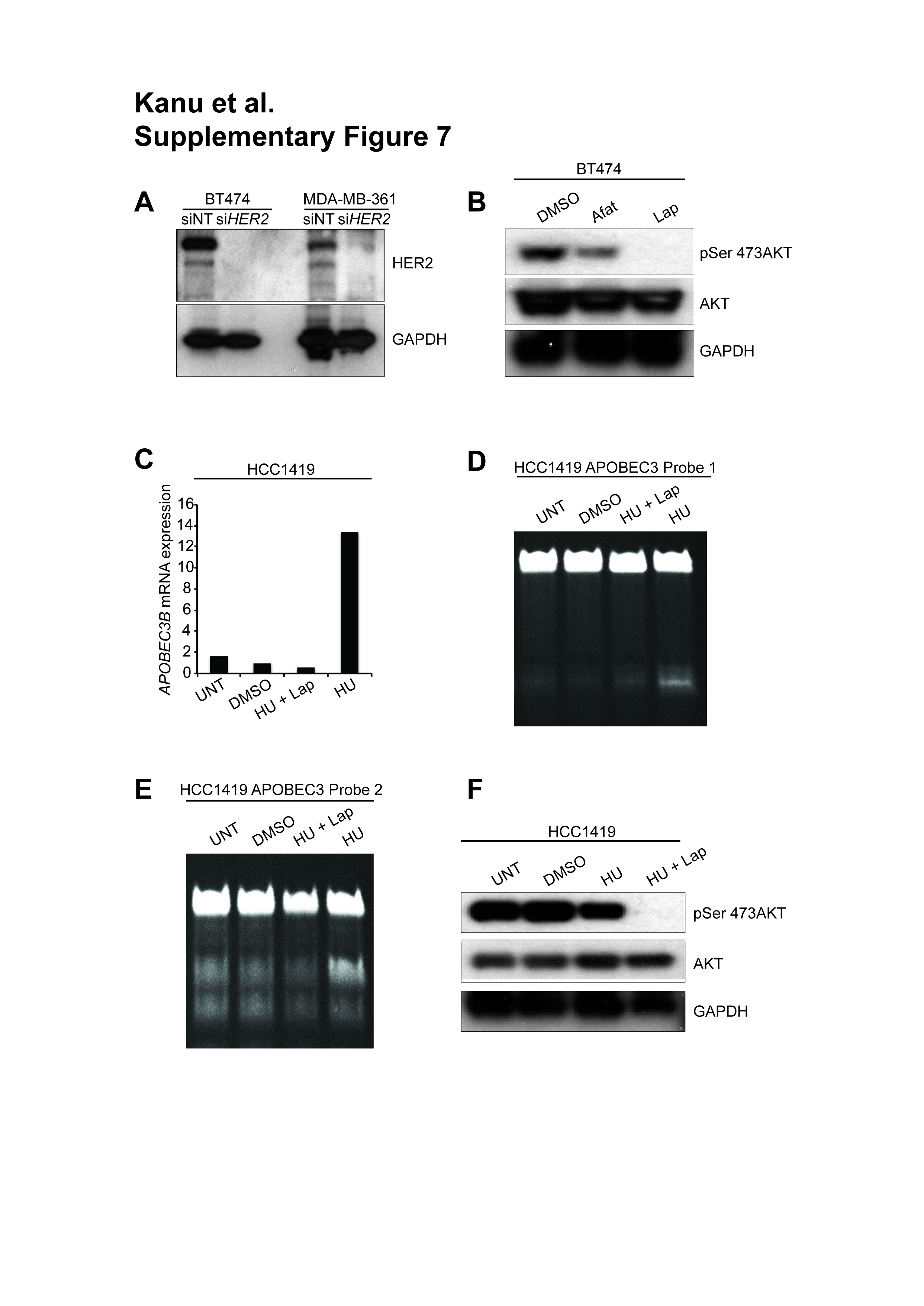

Supplement: Additional file 7: Figure S7. — Role of receptor tyrosine kinase signalling in APOBEC3 activation. a BT474 and MDA-MB-361 cells were treated with RNAi targeting ERBB2. After 72 h, cells were harvested, lysed and western blots were probed with the indicated antibodies to determine the extent of ERBB2 silencing. b BT474 cells were treated with 10 nM afatinib or 30 nM lapatinib for 24 h followed by lysis. Western blots were probed with the indicated antibodies. c HCC1419 cells were treated with 2 mM hydroxyurea in the presence or absence of 30 nM lapatinib. Following mRNA isolation and cDNA synthesis, APOBEC3B mRNA expression levels were determined by quantitative PCR. d HCC1419 cells were treated as in c and, following lysis, oligonucleotide-based cytidine deamination assays were performed for APOBEC3 activity using probe 1. e HCC1419 cells were treated as in c and, following lysis, APOBEC3 cytidine deamination assays were performed using probe 2. f HCC1419 cells were treated as in c. Cells were lysed and western blots were probed with the indicated antibodies. (TIF 34667 kb) [file 13059_2016_1042_MOESM7_ESM.tif]

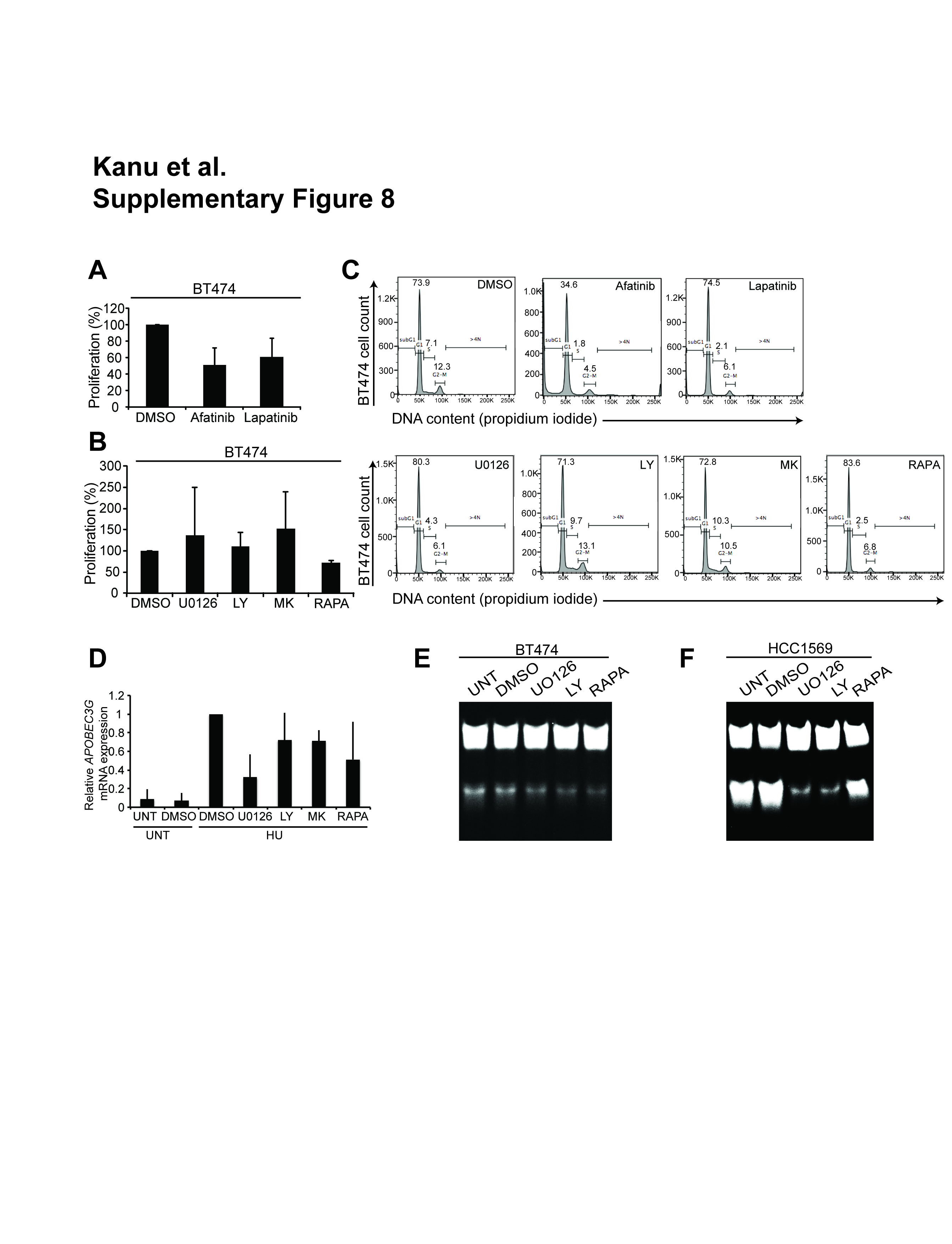

Supplement: Additional file 8: Figure S8. — Role of MAPK and PI3K pathways in APOBEC3 activation. a BT474 cells were treated with 10 nM afatinib or 30 nM lapatinib for 24 h followed by cell viability determination by CellTiter-Glo. b BT474 cells were treated with the indicated drugs for 48 h followed by cell viability determination by CellTiter-Glo. c BT474 cells were treated as in a and b followed by analysis of cell cycle distribution by FACS. d MCF10A cells were treated with the indicated drugs as in Fig. 4g followed by RNA isolation, cDNA synthesis and quantitative PCR to determine APOBEC3G levels. e BT474 cells and f HCC1569 cells were treated with the indicated drugs for 48 h. Cells were lysed and oligonucleotide-based cytidine deamination assays were performed for APOBEC3 activity. (TIF 37363 kb) [file 13059_2016_1042_MOESM8_ESM.tif]

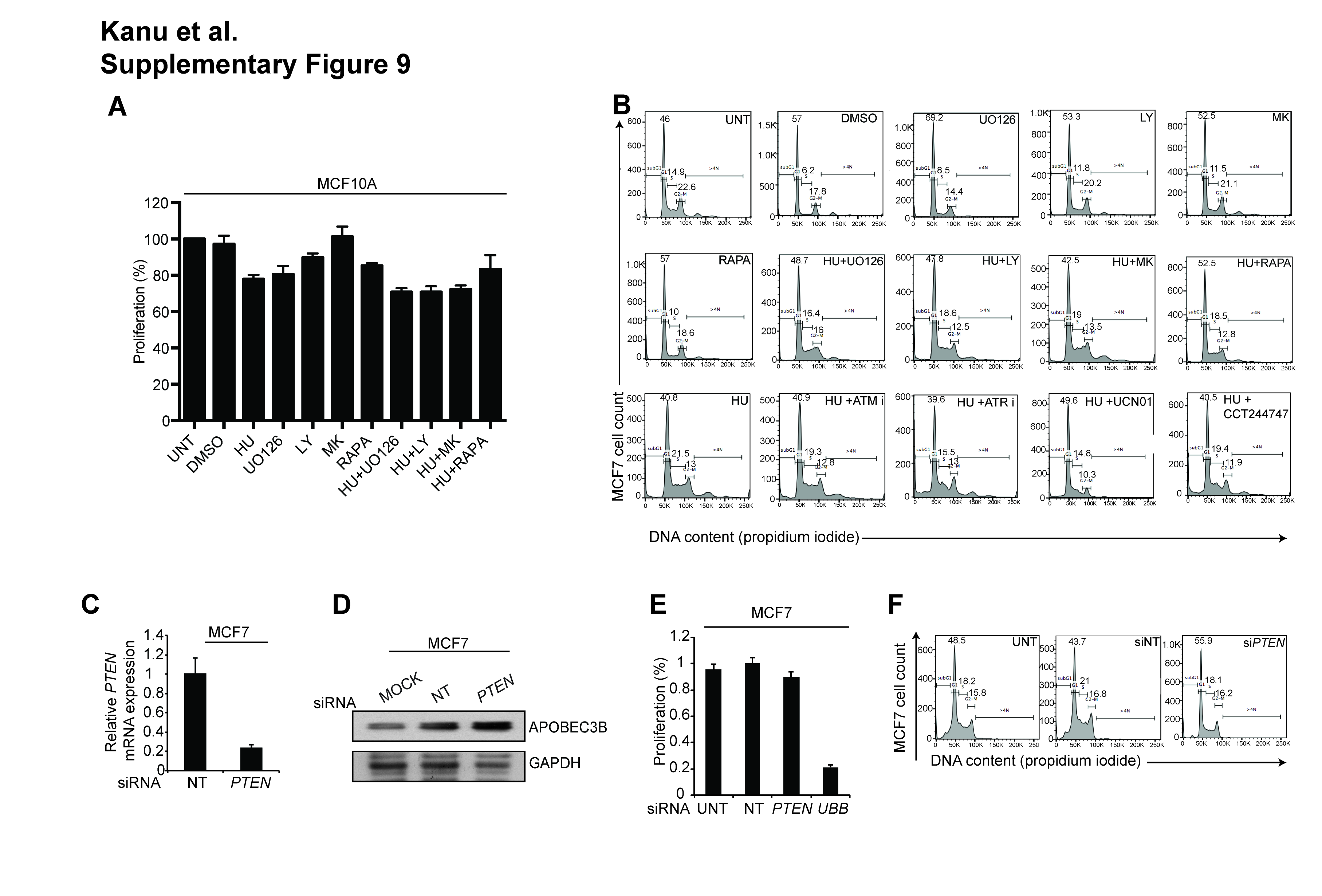

Supplement: Additional file 9: Figure S9. — Role of MAPK and PI3K pathways in hydroxyurea-induced APOBEC3 activation. a MCF10A cells were treated with the indicated drugs for 48 h followed by cell viability determination by CellTiter-Glo. b MCF7 cells were treated with the indicated drugs for 48 h followed by analysis of cell cycle distribution by FACS analysis. c MCF7 cells were transfected with PTEN siRNA. After 72 h the extent of knockdown was determined by quantitative PCR. d PTEN levels were depleted from MCF7 cells by RNAi. After 72 h cells were harvested and western blots were probed with the indicated antibodies. e MCF7 cells were transfected with PTEN siRNA or ubiquitin (UBB) control, followed by cell viability determination by CellTiter-Glo. f MCF7 cells were treated as in c followed by cell cycle distribution analysis by FACS. (TIF 35459 kb) [file 13059_2016_1042_MOESM9_ESM.tif]

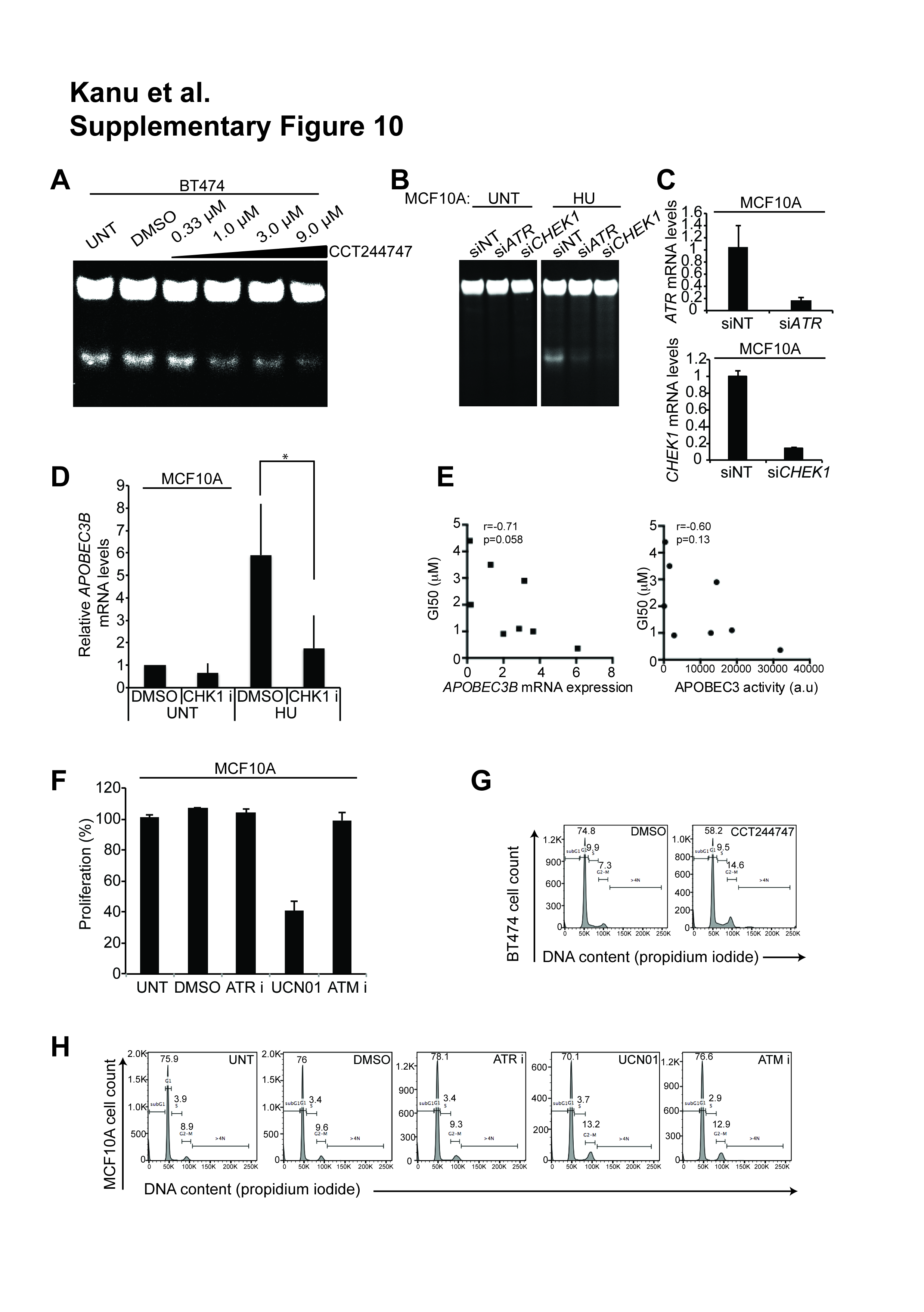

Supplement: Additional file 10: Figure S10. — Role for DNA damage signalling in APOBEC3 activation. a BT474 cells were treated with the indicated doses of Chk1 inhibitor CCT244747 followed by APOBEC3 cytidine deamination assay. b ATR and CHEK1 were depleted from MCF10A cells by RNAi. After 24-h transfection, cells were treated with hydroxyurea for a further 48 h prior to lysis and cytidine deamination assay for APOBEC3 actvity. c Validation of the extent of silencing of ATR and CHEK1 in MCF10A cells. Cells were treated with the indicated siRNAs for 72 h followed by mRNA isolation, cDNA synthesis and quantitative PCR for ATR and CHEK1 mRNA expression levels. d MCF10A cells were treated with hydroxyurea in the presence or absence of Chk1 inhibitor UCN-01 for 48 h prior to mRNA isolation, cDNA synthesis and quantitative PCR for APOBEC3B levels. e Eight breast cancer cell lines were treated with ten doses of CCT244747 for two population doublings followed by sulforhodamine B staining and GI50 determination. f MCF10A cells were treated with the indicated drugs for 48 h followed by cell viability determination using CellTiter-Glo. g BT474 cells were treated with 9 μM CCT244747 followed by analysis of cell cycle distribution by FACS. h MCF10A cells were treated with the indicated drugs for 48 h followed by analysis of cell cycle distribution by FACS. (TIF 34524 kb) [file 13059_2016_1042_MOESM10_ESM.tif]

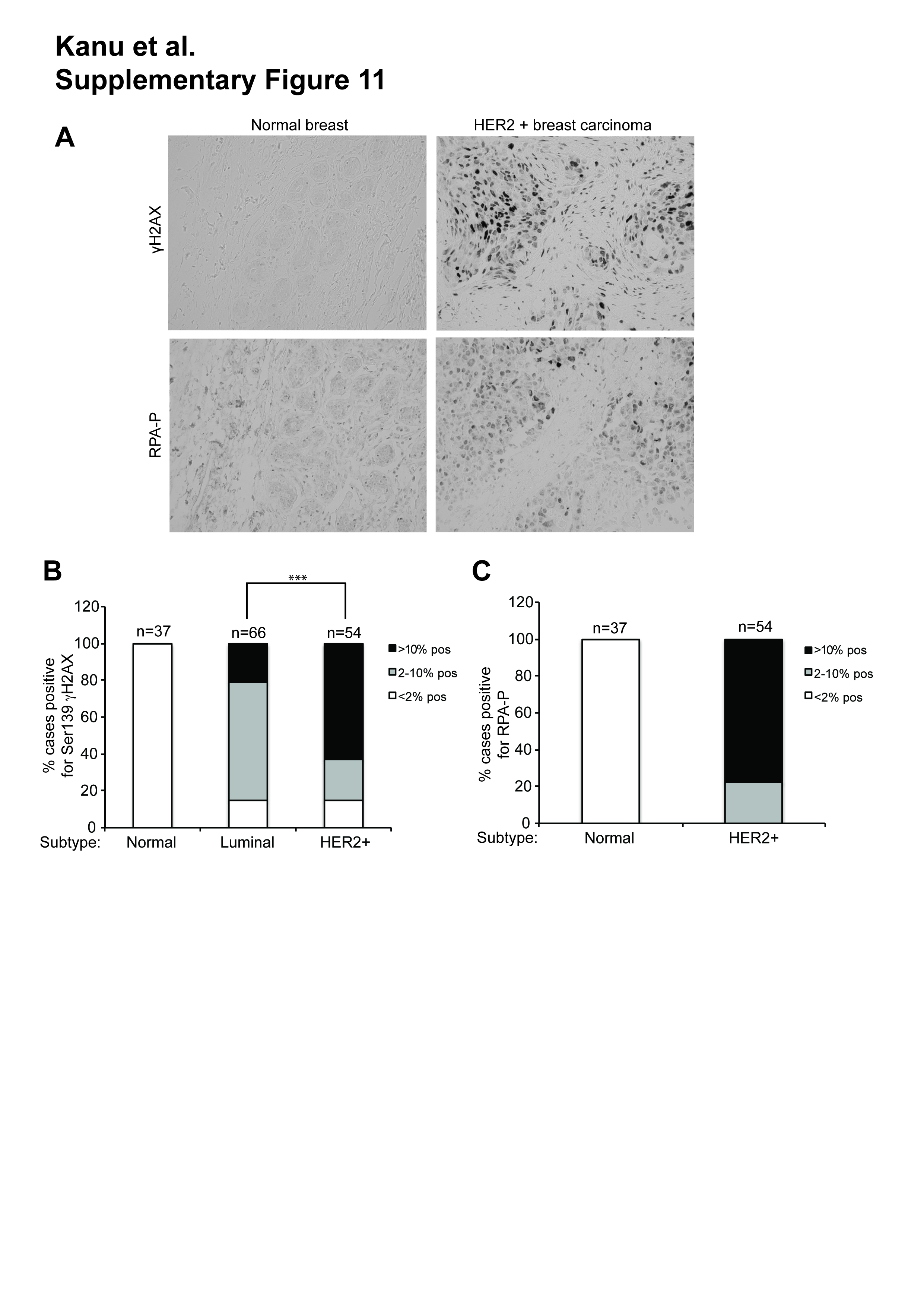

Supplement: Additional file 11: Figure S11. — The HER2+ breast cancer subtype exhibits high levels of γH2AX and RPA-P staining. a Examples of immunohistochemical staining of normal breast and HER2+ tumour samples with Ser139 γH2AX and RPA-P. b The percentages of cases scoring <2 %, 2–10 % and >10 % or more positive for Ser139 γH2AX in each subtype. P = 3.314e-06, Fishers exact test, comparing luminal versus HER2+ subtype exhibiting more than 10 % of cells staining positive for Ser139 γH2AX. c The percentages of cases scoring <2 %, 2–10 % and >10 % or more positive for RPA-P in each subtype. (TIF 34565 kb) [file 13059_2016_1042_MOESM11_ESM.tif]
